# The uncanny valley effect in embodied conversational agents: a critical systematic review of attractiveness, anthropomorphism, and uncanniness

**DOI:** 10.3389/fpsyg.2025.1625984

**Published:** 2025-09-18

**Authors:** Ștefania Cihodaru-Ștefanache, Ioana R. Podina

**Affiliations:** ^1^Laboratory of Cognitive Clinical Sciences, University of Bucharest, Bucharest, Romania; ^2^Interdisciplinary School of Doctoral Studies, University of Bucharest, Bucharest, Romania; ^3^MINDCARE FOR ALL Association, Bucharest, Romania; ^4^Department of Applied Psychology, University of Bucharest, Bucharest, Romania

**Keywords:** Uncanny Valley Effect, embodied conversational agent, systematic review, human-computer interaction, cognition, anthropomorphism

## Abstract

**Introduction:**

The Uncanny Valley Effect (UVE) describes the discomfort users feel when interacting with Embodied Conversational Agents (ECAs) that display human-like features, often resulting in anxiety, disgust, and avoidance. This systematic review investigates how user characteristics and ECA design features influence UVE, aiming to provide insights for improving user engagement.

**Methods:**

Following PRISMA guidelines, we screened 21,897 papers from ACM Digital Library, IEEE Xplore, Scopus, ProQuest, and Web of Science, with 29 studies meeting the inclusion criteria. These studies focused on the roles of anthropomorphism, attractiveness, and uncanniness in user interactions with ECAs.

**Results:**

Using the Effective Public Health Practice Project (EPHPP) tool, most studies were rated as having weak to moderate methodological quality. We developed a Checklist for Avoiding the Uncanny Valley Effect in ECAs, offering critical recommendations across key dimensions such as physical appearance, non-verbal and verbal communication, and the incorporation of social and cultural norms. Additionally, our review underscores the need for methodological improvements.

**Discussion:**

Future studies must address confounding variables with greater precision, provide transparent reporting on participant withdrawal, and employ more robust, standardized measurement tools to generate reliable and actionable findings. Without these advancements, the field risks perpetuating inconclusive and contradictory insights, limiting the development of ECAs that effectively engage users while mitigating the UVE.

**Systematic review registration:**

https://www.crd.york.ac.uk/PROSPERO/view/CRD42023426584, identifier: CRD42023426584.

## Introduction

Embodied Conversational Agents (ECAs) are revolutionizing education and healthcare, bringing cost-effective, adaptable, and portable solutions to the table (Boian et al., 2024; Kavanagh et al., 2017; Philip et al., 2020; Podina et al., 2023; Podina and Caculidis-Tudor, 2023; Ter Stal et al., 2021). In a nutshell, ECAs are digital entities with anthropomorphic features that facilitate both *verbal* and *non-verbal* interactions with users (Liew and Tan, 2021; Loveys et al., 2020a). Their interaction skills are becoming more versatile. ECA can emulate intuitive interactions with people via vocal characteristics, facial expressions, gestures, and, more recently, personality traits (Liew and Tan, 2021; Nass and Moon, 2000; Sebastian and Richards, 2017; Provoost et al., 2017; Ter Stal et al., 2021). In human interactions, research consistently shows that similarity fosters more enjoyable communication and a stronger interpersonal bond (Burleson and Denton, 1992; Philipp-Muller et al., 2020). This principle has influenced the design of ECAs, under the assumption that greater anthropomorphism would lead to more pleasant interaction with ECAs. However, studies have shown a paradoxical effect when it comes to achieving the optimal level of anthropomorphism, known as the *Uncanny Valley Effect* (UVE), which is an intriguing facet of user psychology that remains conceptually and empirically inconsistent.

In this review, we do not aim to suggest a novel definition, further amplifying the lack of consensus in the literature, but rather to clarify existing ones by organizing them within a coherent conceptual model. We adopt a tripartite model of the UVE that distinguishes between three key components and apply this theoretical framework specifically in the case of ECAs: anthropomorphism, attractiveness, and uncanniness (Diel et al., 2021; Ho and MacDorman, 2017; Mara et al., 2022; Mori et al., 2012; Stein and Ohler, 2017; Zhang et al., 2020): (1) *Anthropomorphism* refers to the degree to which an ECA resembles users in terms of physical, behavioral, and mental characteristics, (2) *Attractiveness* is related to the positive appraisal of an ECA, perceived as enjoyable, likeable, intelligent, or friendly, and (3) *Uncanniness* refers to the negative appraisal of an ECA, perceived as disgusting, ugly, or threatening. We broadened the UVE definitions to encompass the emotions and behavioral reactions of the user. Typically, once an ECA’s anthropomorphism increases, the attractiveness also increases until a threshold of around 65% (Slijkhuis, 2017). Heightened levels of attractiveness in ECAs can trigger emotions like calmness, happiness, enthusiasm, and a greater willingness to engage with the ECA (Diel et al., 2021; Ho et al., 2008). However, beyond that threshold of anthropomorphism, attractiveness decreases and uncanniness increases. At higher levels of uncanniness, users experience emotions like fear, anxiety, or disgust and a willingness to avoid the ECA (Mori et al., 2012; Slijkhuis, 2017; Urgen et al., 2018). The exact point where this shift occurs is still debated, with some research suggesting that UVE is strongest when perceived anthropomorphism is between 10 and 30% or 70–90% (Kim et al., 2020; Mori et al., 2012).

Understanding UVE remains challenging due to the existence of multiple competing hypotheses that try to explain our perception of anthropomorphism in ECAs. These range from the morbidity and movement hypotheses to the category ambiguity (Cheetham and Jancke, 2013; Kätsyri et al., 2015; Pollick, 2010). Among these, the perceptual mismatch hypothesis has received strong empirical support. It suggests that users feel uncanniness when they perceive inconsistencies across different levels of anthropomorphism between ECAse features (Kätsyri et al., 2015; Pollick, 2010). Another influential explanation of the UVE is provided by the Cognitive Expectation Violation Theory (CEVT), which proposes that highly anthropomorphic ECAs may generate unrealistic expectations, which, when unmet, lead to uncanniness (Grimes et al., 2021). However, as ECAs become sophisticated, not only in their appearance, but also in their ability to simulate emotions and mental states, appearance-based theories alone no longer suffice. The Uncanny Valley of Mind (UVM) broadens this perspective by highlighting the role of perceived cognitive and emotional anthropomorphism (Desideri et al., 2021; Di Natale et al., 2023; Gray and Wegner, 2012; Stein and Ohler, 2017). Still, determining an excessive level of anthropomorphism remains difficult, as it is not simply an experimental variable to be manipulated in controlled conditions. Anthropomorphism is also a subjective and context-dependent perception shaped by user characteristics (Dubois-Sage et al., 2023). One such characteristic is Theory of Mind (ToM), which is the ability to attribute emotional states to others in order to understand and predict behaviors (Dubois-Sage et al., 2023; Premack and Woodruff, 1978). ToM is linked to social activity and verbal reasoning of the user (Iglesias-Pazo et al., 2025). These individual differences complicate the efforts to predict outcomes such as attractiveness and uncanniness. To better align theory with the features of next-generation ECAs, this systematic review explores how user characteristics and ECA features may mediate or moderate the relationship between anthropomorphism, attractiveness and uncanniness.

Furthermore, the empirical study of the UVE is hampered by methodological inconsistencies, particularly in how the UVE is measured. A major limitation lies in the over-reliance on subjective self-report instruments, which use binary adjective pairs (e.g., *familiar-unfamiliar*, *inert-interactive*) drawn from widely used tools such as the Godspeed Questionnaire (Bartneck, 2023; Ho and MacDorman, 2017; Tobis et al., 2023). However, these scales often lack the nuance needed to capture the emotional ambivalence central to the UVE. Moreover, certain items may be semantically ambiguous: for instance, the term *interactive* might be interpreted as physically responsive by some users and socially communicative by others, undermining reliability and interpretability. Behavioral measures, such as eye-tracking, are also frequently used but raise interpretive challenges. These responses may reflect perceptual salience or cognitive load rather than affective discomfort (Cheetham and Jancke, 2013; Matsuda et al., 2012), making it difficult to isolate the psychological mechanisms specific to UVE. Similarly, although physiological and neural data (e.g., EEG) are occasionally included, no consistent biomarker has been established across studies or stimulus types (Gorlini et al., 2023). Despite the absence of a clear consensus on UVE in the literature, its real-world financial consequences are undeniable. Disney’s infamous $150 million loss from “Mars Needs Moms” due to unsettling character designs is a stark reminder of how the UVE can severely impact humans (Schwind et al., 2018).

The present paper provides a robust evaluation of past research and offers recommendations for future studies, helping scientists and practitioners develop ECAs that effectively mitigate the UVE while enhancing user engagement. Our systematic review goes beyond previous work by simultaneously examining how user characteristics and ECA features interact to shape perceptions of anthropomorphism, attractiveness, and uncanniness. This approach not only clarifies the underlying mechanisms of the UVE but also offers practical insights for designing ECAs that are better aligned with user expectations. Specifically, we address three central research questions to advance the field: Q1. To what extent is UVE present in user interactions with ECAs? Currently, the presence of UVE in ECA interactions remains uncertain. While there is significant evidence of UVE in human-robot interactions, much less is known about its occurrence in user-ECA interactions; Q2. Which user characteristics are associated with how users perceive the ECA in terms of anthropomorphism, attractiveness, or uncanniness? Examining how user characteristics impact perceptions is important for tailoring a customer profile, which allows for more effective interactions; Q3. What ECA features are connected to how users perceive the ECA in terms of anthropomorphism, attractiveness, or uncanniness? Pinpointing which ECA features shape user perceptions enables us to refine design elements and make ECAs more appealing.

This systematic review presents several innovative contributions that address critical gaps in the literature on the UVE. *Firstly*, this review breaks new ground by investigating the behavioral and mental attributes of ECAs that contribute to perceptions of anthropomorphism, attractiveness, or uncanniness—areas that have been largely neglected in favor of a focus on physical appearance (Kätsyri et al., 2015; Mara et al., 2022). Prior studies have disproportionately emphasized the visual resemblance of ECAs to humans, despite evidence that the UVE intensifies when ECAs mimic not just physical traits but also cognitive and emotional characteristics (Jiang et al., 2022; Stein and Ohler, 2017). By shifting the focus to these less explored aspects, this review offers a more nuanced understanding of what makes an ECA feel “human-like” and how this can trigger both positive and negative reactions. *Secondly*, this review is pioneering in its examination of how the UVE may evolve during active user-ECA interactions. Most studies to date have relied on passive forms of engagement, such as showing participants photos or videos of ECAs, which do not fully capture the complexity of real-time interaction (Santamaria and Nathan-Roberts, 2017). An active type of interaction implies dynamic conversational exchanges between users and ECAs. This review offers a more realistic assessment of how the UVE manifests in everyday settings, where users are not merely passive observers but active participants in the interaction. *Finally*, the review goes beyond a purely theoretical contribution by offering both methodological and practical recommendations that can guide future research and development. These insights are designed to help scientists improve experimental designs and assist engineers in creating ECAs that are not only more effective but also tailored to individual user traits. This forward-thinking approach emphasizes the need for personalized ECAs that can better accommodate user diversity, enhancing both usability and emotional engagement. In sum, this systematic review provides a much-needed critical analysis of the UVE, addressing its underexplored aspects and offering actionable solutions.

## Materials and methods

### Search strategy

Potentially relevant papers were found after a thorough search of Scopus, Web of Science, ProQuest, IEEE Explore, and ACM Digital Library in July 2024. These databases were selected based on an initial scan of systematic reviews and meta-analyses on user-ECA interactions (Dey et al., 2018; Diel et al., 2021; Jiang et al., 2022; Kätsyri et al., 2015; Kavanagh et al., 2017; Kim et al., 2020; Liew and Tan, 2021; Yao and Luximon, 2020), which revealed that these five sources were the most commonly used. The search strategy was designed to prioritize recall over precision in the initial phase, aiming to capture a broad, interdisciplinary body of literature on the UVE and ECAs, which resulted in over 21,000 records. We cross-checked our results against reference lists from recent reviews and key studies in th field to ensure adequate coverage.

The full search string is provided below:

("uncanny valley" OR "uncanny valley effect" OR user* OR similar* OR real* OR affinity OR familiar* OR warm* OR likab* OR pleas* OR attract* OR appeal* OR friend* OR natural* OR intelligen* OR esthetic OR beaut* OR harm* OR accept* OR valence OR arousal OR eerie OR creep* OR uncann* OR weird OR strange* OR typic* OR comfort* OR threat* OR dominan* OR ugl* OR dull OR freak* OR predict* OR bor* OR shock* OR thrill* OR bland OR emotional OR anomaly OR disgust*) **AND** (embodied agent* OR embodied conversation* agent OR embodied conversation* OR interface agent* OR embodied social agent* OR embodied virtual agent* OR embodied companion agent* OR embodied computer agent* OR relational agent* OR empathic agent* OR conversation* agent* OR interface agent* OR animated agent* OR computer agent* OR emotion agent* OR exercise agent* OR motivation* agent* OR virtual agent* OR virtual character* OR virtual user* OR virtual coach* OR virtual advisor* OR virtual specialist* OR virtual dialog* agent* OR avatar OR pedagogical agent* OR learning partner* OR virtual tutor* OR social robot*) **AND** (experience* OR user* OR expectation* OR usability OR understanding* OR bias* OR emotion* OR attitude* OR interact* OR conversation* OR cooperat* OR cognit* OR evaluation OR assessment OR social*).

The search string was meticulously constructed by employing previously defined synonyms for the UVE (Diel et al., 2021; Zhang et al., 2020), synonyms for the ECA (Loveys et al., 2020a), and for user-chatbot interactions (Rapp et al., 2021).

### Inclusion and exclusion criteria

We included research that assessed a minimum of (a) one of the UVE variables (anthropomorphism, attractiveness, or uncanniness), through (b) quantitative data based on (c) dynamic and engaging interactions involving dialogues or interactive gaming experiences (d) between individuals and ECAs. Specifically, we focused on papers that examined (e) how users perceive social interaction with (f) ECA representations that differ from the physical characteristics of the individuals involved. These studies were required to be (g) peer-reviewed and written in (h) English. Finally, the age of the participants wasn’t an inclusion criterion.

We excluded qualitative research without reported data, as well as studies involving individuals with psychological or physical disabilities, such as autism spectrum disorder, dementia, or multiple sclerosis, as the perception of the ECAs might differ (Feng et al., 2018; Olaronke et al., 2017). Moreover, we also excluded studies examining interactions with ECA through images or videos, since they can be considered passive forms of interaction (Coan and Allen, 2007), especially due to the ECA’s inability to respond to user input. Additionally, we excluded research that focused solely on ECA design and development or user performance in a task. Furthermore, we excluded research featuring ECAs with machine-like or pet-like appearances, as these features are expected to lower perceived uncanniness reported by users (MacDorman, 2005). Finally, studies where ECAs shared the same face or body as participants were also excluded, as this choice of representation might lead to higher uncanniness regarding the ECA (Schwind et al., 2017).

### Selection of studies

The review protocol has been officially registered on PROSPERO^1^ under the registration number: CRD42023426584. This review followed the guidelines outlined in the Preferred Reporting Items of Systematic Reviews and Meta-Analyses^2^.

**Figure 1:**
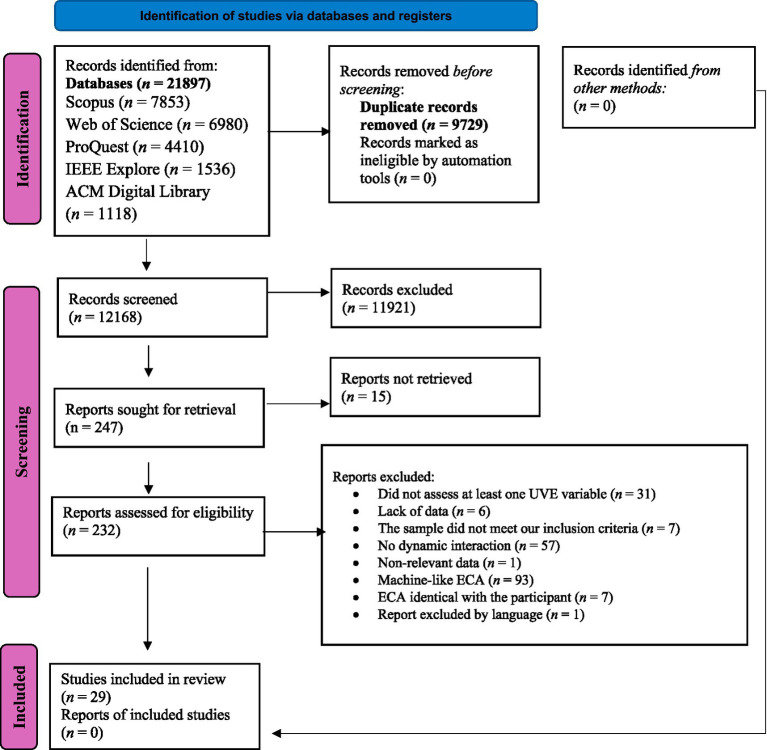
PRISMA flow diagram of the selection process.

Following an exhaustive search, we initially identified a total of 21,893 online records, as depicted in Figure 1. After removing duplicates, we examined the title and abstracts of the remaining studies to assess their potential relevance. The full text of the remaining 247 articles was analyzed in detail. Our meticulous selection process resulted in the inclusion of 29 studies that rigorously met the predefined criteria. The citations for these included publications are accessible in the Supplementary materials.

### Data extraction

Table 1 presents the key characteristics of the included studies. Data extraction was guided by a standardized coding scheme developed based on prior reviews (Liew and Tan, 2021; Loveys et al., 2020b) and structured around the PEO model (Population–Exposure–Outcome), which is widely used in systematic reviews to enhance methodological transparency (Hosseini et al., 2024). Additionally, while we calculated inter-rater agreement for the quality appraisal of the included studies, we did not conduct inter-rater reliability procedures during the data extraction phase. Data extraction was performed by one author, with ongoing consultation and consensus discussions with a senior co-author. Nevertheless, the absence of independent double coding means that some degree of individual bias cannot be entirely ruled out, despite our best efforts to ensure accuracy and consistency. To ensure that the template for data extraction captured all relevant information, we piloted it on two studies, as recommended in the best practice guidelines (Büchter et al., 2020; Higgins and Green, 2008). Any discrepancies or uncertainties were discussed and resolved by consensus.

**Table 1:** Characteristics of the studies included.

References	UVE Status	ECA type (gender, body motion)	User-ECA interaction			Study characteristics			
			ECA behavior type (scripted, wizard of Oz, autonomous)	User characteristics (age range, mean age, standard deviation)	Type and time of engagement	Sample size (% females)	Randomization	UVE outcomes (assessment method)	Assessment tools (Nr. of items)
Appel et al. (2012)	Not clear	Female, Half body, Dynamic	NS	Mixed ages, M = 36.2, SD = 12.2	Structured dialogue, NS	90 (54%)	Yes	Attractiveness, Uncanniness (Subjective, Behavioral)	RS (24)
Bailey and Schloss (2024)	Yes	Female, Full Body, Dynamic	NS	Children, M = 7.92, SD = 1.1	Game, 20 min	25 (32%)	Yes	Anthropomorphism, Uncanniness (Subjective, Behavioral)	IDAQ-CF (12)
Belda-Medina and Calvo-Ferrer (2022)	Not clear	Female, Half body, Dynamic	Scripted	Young adults, NS, NS	Training, NS	176 (80%)	No	Attractiveness (Subjective, Behavioral)	CHISM (15)
Buttussi and Chittaro (2019)	Not clear	Male, Half body, Dynamic	Autonomous	Young adults, M = 22.0, SD = 1.7	Training, 30–45 min	94 (20%)	No	Attractiveness, Uncanniness (Subjective)	APAPQ (26)
Conrad et al. (2015)	Yes	Male, Face only, Dynamic	Scripted	Mixed ages, M = 36.8, NS	Structured dialogue, 5–7 min	75 (50%)	Yes	Attractiveness Uncanniness and Anthropomorphism (Subjective, Behavioral)	NA (11)
Creed and Beale (2012)	Not clear	Female, Face only, Dynamic	Scripted	Young adults, NS, NS,	counseling, 10 min	50 (58%)	Yes	Attractiveness Uncanniness (Subjective)	NA (24)
Falcone et al. (2022)	Not clear	Female, Half body, Dynamic	Wizard of Oz and Scripted	Mixed ages, NS, NS	Structured dialogue, 5 min	36 (44%)	No	Attractiveness, Anthropomorphism (Subjective)	GQS (NS)
Hale and Hamilton (2016)	Not clear	Female, Face only, Dynamic	NS	NS, NS, NS	Structured dialogue, NS	54 (68%)	No	Attractiveness (Subjective, Behavioral)	NA (1)
Ham et al. (2024)	Not clear	Female, Half body, Static	NS	Mixed ages M = 31.07, SD = 5.71	Instagram posts, 0.16 min	165 (44%)	Yes	Anthropomorphism, Attractiveness, Uncanniness (Subjective)	PEI, PA, ATVI (45)
Hao et al. (2024)	Not clear	Female, Half body, Dynamic	Scripted	Mixed Ages, NS NS	Structured dialogue, NS	354 (32%)	Yes	Attractiveness (Subjective)	NA (1)
Lahav et al. (2020)	Not clear	Female, Half body, Dynamic	NS	Young adults, M = 23.24, SD = 2.28	counseling, 12 min	42 (57%)	No	Attractiveness, Uncanniness (Subjective)	NA (2)
Lisetti et al., 2004	Not clear	Female, Face only, Dynamic	NS	Young adults, M = 23.04, SD = 3.11	Structured dialogue	56 (75%)	No	Attractiveness, Anthropomorphism (Subjective)	NA (8)
Luo et al. (2023)	Not clear	Mixed, Half body, Dynamic	NS	Young adults, M = 23.77, SD = 2.95	Game, NS	48 (56%)	Yes	Attractiveness, Uncanniness (Subjective)	IEPS (8)
Min et al. (2024)	Not clear	Mixed, Half body, Dynamic	Scripted	NS, NS, NS	Structured dialogue, NS	465 (63%)	Yes	Attractiveness (Subjective)	NA (4)
Neumann et al. (2023)	Not clear	Female, Full body, Dynamic	NS	Young adults, M = 24.47, SD = 4.45	Structured dialogue, 25 min	36 (58%)	Yes	Anthropomorphism (Subjective)	NA (2)
Prendinger and Ishizuka (2001)	Not clear	Male, Full body, Dynamic	Scripted	NS, NS, NS	Structured dialogue, 3 min	16 (NS)	Yes	Attractiveness (Subjective)	NA (1)
Prendinger et al. (2006)	Not clear	Male, Face only, Dynamic	NS	Mixed ages, M = 30, NS	Game, 10 min	32 (56%)	Yes	Uncanniness	NA (NA)
Saad and Choura (2022)	Not clear	NS, NS, Dynamic	NS	Mixed ages, NS, NS,	Structured Dialogue, NS	1,262 (41%)	No	Anthropomorphism (Subjective)	PRS (12)
Sajjadi et al. (2019)	Not clear	Female, Half body, Dynamic	Scripted	Mixed ages, NS, NS,	Structured Dialogue, 15 min	41 (46%)	Yes	Uncanniness, Attractiveness (Subjective)	GEQ (3)
Schouten et al. (2017)	Not clear	Female, Half body, Static	Wizard of Oz	Mixed ages, M = 25.7, SD = 4.4	counseling, 30 min	34 (41%)	No	Attractiveness (Subjective, Behavioral, Physiological)	PAQ (3)
Song and Shin (2022)	Yes	Male, Face only, Mixed	NS	Young adults, M = 24.2 SD = 6.2,	Structured Dialogue, 0.75 min	185 (63%)	Yes	Uncanniness (Subjective)	SHM (4)
Ter Stal et al. (2021)	Not clear	Female, Half body, Dynamic	Wizard of Oz and Autonomous	Mixed ages, M = 48, SD = 22	Structured Dialogue, NS	63 (57%)	Yes	Anthropomorphism, Attractiveness (Subjective)	RS (3)
van Pinxteren et al. (2023)	Not clear	Female, Half body, Dynamic	Scripted	Mixed ages, M = 23, SD = 6.5	Structured Dialogue, NS	142 (63%)	Yes	Attractiveness (Subjective)	SFVDP (10)
Volante et al. (2016)	Yes	Male, Half body, Dynamic	NS	Young adults, NS, NS	Training, NS, NS	62 (41%)	No	Attractiveness, Uncanniness (Subjective)	NA (3)
Wang et al. (2021)	Yes	Female, Half body, Dynamic	NS	Mixed ages, M = 25.3, SD = 7.4	Game, 2 min	21 (42%)	Yes	Anthropomorphism, Attractiveness (Subjective)	NA (4)
Yin et al. (2021)	Yes	Male, Face only, Static	NS	Young adults, NS, NS	Structured Dialogue, 4 min	80 (53%)	Yes	Anthropomorphism, Uncanniness (Subjective)	NA (4)
Zhang et al. (2024)	Not clear	Female, Half body, Static	NS	Young adults, M = 26.12 NS	Structured Dialogue, NS	183 (59%)	Yes	Anthropomorphism (Subjective)	NA (4)
Zheleva et al. (2023)	Yes	Female, Half body, Dynamic	Scripted	NS, M = 25.6, SD = 11.3	Game, NS	44 (100%)	No	Uncanniness (Subjective, Behavioral)	GQS (1)
Zibrek et al. (2018)	Yes	Male, Mixed, Dynamic	NS	NS, NS, NS	Structured Dialogue, NS	222 (NS)	Yes	Anthropomorphism, Attractiveness, Uncanniness Subjective	NA (5)

NS, Not specified; NA, Not applicable; RS, Rapport Scale; CHISM, Chatbot-User Interaction Satisfaction Model; APAPQ, Animated Pedagogical Agent Perception Questionnaire; GQS, Godspeed Questionnaire Series; PRS, Perceived Realism Scale; GEQ, Game Experience Questionnaire; PAQ, Participant Assessment Questionnaire (affective subscale); SHM, Scale proposed by Ho and MacDorman (subscale Uncanniness); RS, Rapport Scale; IDAQ-CF, The Individual Differences in Anthropomorphism-Child Form; PEI, Perceived emotional intelligence; PA, Perceived anthropomorphism; ATVI, Attitude toward the Virtual Influencer; IEPS, Izard Emotional Perception Scale; SFVDP, scale from Von Der Pütten et al. (2010). Rows highlighted in green indicate significant evidence for the Uncanny Valley Effect. Rows highlighted in yellow indicate inconclusive results regarding the Uncanny Valley Effect.

*First*, we extracted publication details, including the author(s) details and year of publication. *Second*, population characteristics such as total sample size, gender distribution, and average age with standard deviations. *Third*, we extracted information about the exposure to ECAs, including whether the study used a randomized or non-randomized design, ECA Behavior Type (Scripted, Wizard of Oz, Autonomous), the ECA’s gender, body type (e.g., face-only, half-body without legs, or full-body with legs), type of motion (e.g., static, capable of gestures, or full-body movement), and time of exposure (in minutes). We also examined the type of engagement involved, specifically what the users and ECAs did during the interaction. Here, we differentiated between simple scripted conversations (e.g., structured dialogues), more complex interactions requiring adaptability from the ECA, such as counseling (e.g., for health-related guidance) or training (e.g., educational tasks). *Finally*, we coded the outcomes assessed, specifying the type and number of outcome measures used. We differentiated between subjective ratings (e.g., Godspeed indices), behavioral responses (e.g., reaction times), and physiological measures (e.g., EEG). We also noted whether measurement tools were standardized or developed *ad hoc*, and whether the study reported significant results related to the Uncanny Valley Effect (UVE).

### Quality assessment

The assessment of the risk of bias and overall quality of the included studies was performed using the Effective Public Health Practice Project (EPHPP) guidelines (Armijo-Olivo et al., 2014, Sievers et al., 2018). While UVE is not traditionally a public health topic, many of the psychological and emotional factors explored in UVE research overlap with public health studies, particularly in terms of understanding human behavior and wellbeing. The decision to use EPHPP in the present systematic review was based on its versatility in evaluating various study designs and offering a structured approach to judging evidence quality.

The EPHPP guidelines provide a consistent, and comprehensive framework of critical methodological aspects of each study across several dimensions: (a) selection bias, (b) research design, (c) controlling for confounders, (d) blinding, (e) data collection methods, and (f) withdrawals and dropouts (Thomas et al., 2004).^3^ Two independent evaluators assessed each criterion as either strong, moderate, or weak, resulting in an overall quality rating for each study. Each reviewer received identical training and guidance documents for utilizing the tools, ensuring uniformity in their approach. A study was categorized as strong if it received at least four strong ratings and no weak ratings. Studies with less than four strong ratings and no more than one weak rating were assigned a moderate overall rating, whereas studies with at least two weak ratings were classified as weak overall quality (Sievers et al., 2018). The inter-reviewer agreement was strong, with a coefficient of *k* = 0.85 for the overall study quality. Any disagreements were resolved through discussion until a complete consensus was reached. A study classified as strong indicates a lower risk of bias, whereas a study coded as weak suggests a higher risk of bias (see Table 2).

**Table 2:** Quality assessment results following the usage of the EPHPP instrument.

Study ID	Selection	Design	Confounders	Blinding	Data collection	Withdrawals	OVERALL
Appel et al. (2012)	Weak	Strong	Weak	Strong	Strong	Weak	Weak
Bailey and Schloss (2024)	Moderate	Strong	Weak	Moderate	Weak	Strong	Weak
Belda-Medina and Calvo-Ferrer (2022)	Moderate	Weak	Moderate	Moderate	Strong	Weak	Moderate
Buttussi and Chittaro (2019)	Moderate	Strong	Weak	Strong	Strong	Moderate	Moderate
Conrad et al. (2015)	Weak	Strong	Moderate	Strong	Weak	Strong	Weak
Creed and Beale (2012)	Moderate	Strong	Weak	Strong	Moderate	Moderate	Moderate
Falcone et al. (2022)	Moderate	Moderate	Weak	Moderate	Moderate	Weak	Weak
Hale and Hamilton (2016)	Moderate	Moderate	Moderate	Strong	Strong	Strong	Moderate
Ham et al. (2024)	Moderate	Strong	Weak	Moderate	Strong	Weak	Weak
Hao et al. (2024)	Moderate	Strong	Weak	Moderate	Moderate	Weak	Weak
Lahav et al. (2020)	Moderate	Weak	Moderate	Moderate	Strong	Moderate	Moderate
Lisetti et al., 2004	Moderate	Moderate	Weak	Moderate	Strong	Weak	Weak
Luo et al. (2023)	Moderate	Strong	Moderate	Weak	Weak	Weak	Weak
Min et al. (2024)	Moderate	Strong	Strong	Weak	Weak	Moderate	Weak
Neumann et al. (2023)	Moderate	Strong	Weak	Weak	Weak	Weak	Weak
Prendinger and Ishizuka (2001)	Weak	Strong	Weak	Moderate	Moderate	Weak	Weak
Prendinger et al. (2006)	Weak	Strong	Weak	Moderate	Strong	Weak	Weak
Saad and Choura (2022)	Moderate	Strong	Weak	Moderate	Strong	Moderate	Moderate
Sajjadi et al. (2019)	Strong	Strong	Strong	Moderate	Weak	Weak	Weak
Schouten et al. (2017)	Moderate	Moderate	Weak	Moderate	Strong	Weak	Weak
Song and Shin (2022)	Moderate	Strong	Weak	Strong	Strong	Strong	Moderate
Ter Stal et al. (2021)	Weak	Strong	Weak	Strong	Moderate	Moderate	Weak
van Pinxteren et al. (2023)	Moderate	Strong	Strong	Weak	Strong	Strong	Moderate
Volante et al. (2016)	Moderate	Strong	Moderate	Moderate	Moderate	Weak	Moderate
Wang et al. (2021)	Moderate	Moderate	Strong	Moderate	Strong	Strong	Moderate
Yin et al. (2021)	Moderate	Strong	Weak	Weak	Weak	Strong	Weak
Zhang et al. (2024)	Moderate	Strong	Weak	Moderate	Moderate	Weak	Weak
Zheleva et al. (2023)	Moderate	Moderate	Moderate	Moderate	Moderate	Moderate	Moderate
Zibrek et al. (2018)	Moderate	Strong	Weak	Strong	Moderate	Moderate	Moderate

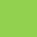
, Strong quality; 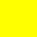, Moderate quality; 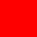, Weak quality.

## Results

### Study characteristics

A substantial portion of the studies included in our systematic review have been published in recent years. The trend in this research area spans over two decades. Notably, up to five studies (17%) were published in 2024, and four studies (13%) were published in 2023, which demonstrates the current importance of the UVE.

The present systematic review encompassed a total of 4,153 users, exhibiting a broad age range from 5 to 88 years old, with a slightly higher representation of females. Regarding ECA features, many studies opted for female-gendered ECAs (18 papers, 62%), and half-body ECAs (17 papers, 58%). The predominant choice among the studies was three-dimensional ECAs exhibiting dynamic movement capabilities (24 papers, 82%) and lacking customization options. More than half of the included studies did not clearly specify what type of behavior had the ECA (16 papers, 55%), but most of the studies that clearly specified it, used ECAs with a scripted behavior. Finally, when it comes to interaction, the average user-ECA interaction duration across studies was 11 min. Unfortunately, most of the studies did not specify the exact engagement time between users and ECAs. Notably, all participants were actively engaged in the interaction with the ECAs. The primary user engagement observed was structured dialogue (17 papers, 58%), followed by interactive games involving both users and ECAs (5 papers, 17%). The remaining studies employed ECA-delivered training sessions (3 papers, 10%), and counseling sessions facilitated by the ECA (3 papers, 10%).

Shifting the focus to the characteristics of the included studies are presented in Table 1. In terms of *study characteristics*, the prevailing design among the included papers was between-subject (18 papers, 62%), followed by within-subject design (7 papers, 24%), and cross-sectional design (3 papers, 15%). Only one study employed a within-subject design (1 paper, 5%). No study within our review explored the UVE in user-ECA interactions through a longitudinal design, tracking changes over time. Notably, more than half of the included studies randomized participants between conditions (19 papers, 65%). Moreover, a minority of studies measured simultaneously all 3 outcomes of the UVE (3 papers, 15%), and the most extensively studied outcome among these papers was the attractiveness of the ECA (21 papers, 72%). All studies utilized subjective measurements (29 papers, 100%), evaluating UVE through questionnaires or single-itemrevisi questions. However, some studies also used behavioral (7 papers, 24%), including metrics such as gaze time or the count of user-initiated interactions. Additionally, a smaller portion utilized physiological measures (1 paper, 3%), employing metrics such as skin conductance. Half of the studies created their own subjective measurements for the UVE with either singular or multiple items (15 papers, 52%), while the remaining studies used questionnaires to measure the UVE outcomes (14 papers, 48%).

Interestingly, a diverse range of data collection techniques was observed, including behavioral measures like word count, usage of pause-fillers (e.g., “erm,” “hm”), frequency of broken words (e.g., “I was in the bib… library”), time spent interacting with the ECA, and gaze time, as well as acknowledgement through channel utterances such as “okay,” “all right,” “got it,” “thank you” (Appel et al., 2012; Bailey and Schloss, 2024; Buttussi and Chittaro, 2019; Min et al., 2024). Additionally, physiological measures including skin conductance, electromyography, and photoplethysmography were employed in some studies (Lahav et al., 2020; Min et al., 2024). Additionally, several studies in our review included qualitative interviews. These interviews aimed to gather in-depth user feedback, asking questions such as: “What did you like most about the ECA?” and “What did you like least about the ECA?” (Volante et al., 2016). This qualitative approach provided valuable insights into user preferences and experiences with the ECA.

### Methodological quality of included studies

A quality appraisal using the EPHPP tool (Armijo-Olivo et al., 2014) revealed that most of the included studies were rated as either *weak* (17 studies, 59%) or *moderate* (12 studies, 41%) in overall methodological quality (see Table 2). This indicates a high risk of bias across the evidence base, limiting the reliability and generalizability of findings related to the UVE.

To begin, a notable strength was that most studies (21 studies, 72%) reported using randomized designs and therefore received a *strong* rating for study design. However, few studies clearly described the randomization process, such as how the allocation sequence was generated and whether allocation was concealed. Without this information, the risk of selection bias remains, despite claims of randomization. Moreover, no studies reported whether randomization accounted for relevant sample characteristics (e.g., gender, age, familiarity with technology), which are likely to influence user responses to ECAs.

In terms of data collection methods, fewer than half of the studies (13 studies, 44%) employed established instruments such as the Godspeed scale. While this tool has its own limitations, it is nonetheless a recognized standard in the field. In contrast, a substantial number of studies relied on *ad hoc* instruments, meaning that items or scales were created specifically for a single study without prior validation or theoretical grounding. Moreover, a particularly concerning issue is the widespread use of subjective rating scales without reporting internal consistency (e.g., Cronbach’s alpha) or construct validation procedures. The lack of psychometrically proven tools seriously undermines the interpretability and comparability of outcomes.

Most studies were rated as *moderate* in terms of participant selection (23 studies, 79%), primarily due to unclear recruitment procedures and limited information on sampling frames. Although many studies used appropriate populations (e.g., adults interacting with ECAs), the absence of details on consent processes, recruitment settings, and inclusion/exclusion criteria limits generalizability and replicability.

Blinding was inconsistently addressed. While over half of the studies mentioned participant blinding (16 papers, 55%), few provided information about whether evaluators or technical personnel were blinded to the study hypotheses or conditions. This omission is especially problematic for studies relying on behavioral responses, where observer bias and expectancy effects can influence results.

Two domains were consistently *weak*: confounder variables (18 papers, 62%) and the withdrawal of participants (14 papers, 48%). Across studies, control for potential confounding variables was generally inadequate. Few investigations accounted for individual differences likely to modulate the UVE, such as prior exposure to ECAs or baseline trait anxiety. The omission of these variables limits the ability to interpret whether observed effects are attributable to the experimental manipulations or to uncontrolled participant characteristics.

Similarly, reporting on participant attrition was often insufficient. In nearly half of the studies, dropout rates were either missing or superficially addressed, leaving it unclear whether participants could withdraw due to technical issues, lack of engagement, or the UVE. Without transparent documentation of participant flow and reasons for withdrawal, it is difficult to assess whether the final samples remained representative of the target population.

### Main results

Half of the studies measured user-ECA engagement through structured dialogue, where ECAs asked questions like “How can I assist you?” or interacted with users by administering surveys with questions related to housing or jobs. Almost all ECAs were dynamic, utilizing gestures or facial expressions. Gender representation was balanced, with half of the ECAs depicted as female and the other half as male, and approximately 50% featured half-body representations. The sample sizes in the studies varied, ranging from 21 to 222 participants, predominantly younger, mixed-gender individuals.

#### Examination of user characteristics related to the UVE outcomes

In our systematic review, a limited number of studies (6 papers, 20%) investigated the role of user characteristics in interactions between users and ECAs (see Table 3). Notably, gender of the users emerged as a key sociodemographic factor (4 papers, 13%), indicating that females generally perceive ECAs as more attractive than males. Female users generally exhibited higher levels of empathy and reported less tension and annoyance toward the ECA (Belda-Medina and Calvo-Ferrer, 2022; Lahav et al., 2020; Lisetti et al., 2004; Sajjadi et al., 2019). Age also played a significant role, with younger participants finding ECAs more attractive (Lisetti et al., 2004; Zhang et al., 2024). Another noteworthy factor is the flow state, which appears when users become deeply immersed and fully engaged in an interaction with the ECA, experiencing a high level of focus and reduced awareness of time or external distractions. Specifically, the flow state of the users positively predicted the perceived anthropomorphism of the ECA in one study rated with a moderate overall methodological quality (Saad and Choura, 2022). Despite similar access to technology, Polish users reported more positive attitudes and a greater perceived ease of use toward ECAs for learning compared to Spanish users, suggesting that ease of use may be linked to overall user attitudes. One possible explanation for the less positive attitudes among Spanish participants is their broader familiarity with advanced conversational agents like Alexa, Siri, Cortana, Google Assistant, and Watson. This familiarity may lead to higher expectations and quicker disappointment due to the habituation effect, reducing curiosity and novelty during interactions with simpler ECAs (Belda-Medina and Calvo-Ferrer, 2022). Interestingly, students from the Faculty of Social Sciences perceived the ECA delivering career counseling as more attractive and were more likely to recall its recommendations, compared to students from the Faculty of Exact Sciences. One possible explanation given by authors is that students in Exact Sciences may have less time or interest in engaging with such activities outside their core studies (Lahav et al., 2020). A research investigation focused on how user personality traits, as characterized by the Big Five Model, affected perceived attractiveness (Lisetti et al., 2004). Participants exhibiting higher levels of openness to experience tended to find the ECA more attractive (Lisetti et al., 2004). However, the study was rated with a weak overall methodological quality.

**Table 3:** Evidence status summary of included studies in the systematic review.

Factor	Main finding	Outcome
User characteristics		
Gender	Female participants showed greater interest in customizing the ECA’s features compared to male participants (Belda-Medina and Calvo-Ferrer, 2022). In other studies, female users were particularly more satisfied with the visual appearance of the ECA and reported significantly more that they would recommend the ECA compared to males (Lahav et al., 2020; Lisetti et al., 2004). Lastly, female users generally exhibited higher levels of empathy and reported less tension and annoyance toward the ECA compared to male users (Sajjadi et al., 2019).	↑ Attractiveness
Education background	When asked if they would take a recommendation from their interactions with ECA into the future, users in social sciences and humanities were significantly more likely to respond positively compared to users in exact sciences, medicine, and engineering (Lahav et al., 2020).	↑ Attractiveness
Personality	Openness to experience was a personality trait that was associated with the higher perceived attractiveness of ECA. Users more open to experience had a more positive view of the ECA than those less open to experience (Lisetti et al., 2004).	↑ Attractiveness
Age	Younger participants perceived the ECA as more attractive (Lisetti et al., 2004; Prendinger et al., 2006).	↑ Attractiveness
Flow	Users who experienced a flow state, characterized by deep involvement in an activity, reported a positive impact on their perception of the ECA’s realism (Saad and Choura, 2022).	↑ Anthropomorphism
Expectations	Users had significantly higher affective expectations of the high anthropomorphic ECA compared to the low anthropomorphic ECA. They anticipated that the ECA would better understand and respond to their emotions (Yin et al., 2021). Therefore, the more the ECA expresses concern or care, the less likely it is to violate the user’s expectations. This relationship was even stronger for ECAs that are highly anthropomorphic, as users tend to expect ECA to be more (Yin et al., 2021).	↑ Attractiveness ↑ Uncanniness
Social norms	Uncanniness did not significantly predict a reduction in eye gaze during user-ECA interactions. Even when users perceived the ECA as uncanny, they continued to maintain eye contact, suggesting that the sense of uncanniness does not strongly disrupt social norms (Zheleva et al., 2023).	↑ Uncanniness
Exposure	Users were more accepting of ECAs displaying emotions after interacting with the ECA than they were beforehand. Specifically, their acceptance of ECAs showing positive emotions increased, and they also became more comfortable with an ECA expressing frustration or anger when faced with obstacles (Luo et al., 2023).	↑ Attractiveness
Familiarity with the ECA	The ECA with a celebrity appearance in a hyper-realistic condition was perceived as less uncanny compared to the ECA with a cartoonish appearance (Song and Shin, 2022).	↑ Uncanniness
Proximity	No significant association was found between perceived proximity to the ECA and feelings of uncanniness. Notably, participants found the ECA interesting and came closer to investigate the details of the ECA.	↑ Uncanniness
ECA features		
Social cues	ECA featuring eye blinking, breathing, posture shifts, and head nods resulted in more favorable perceptions and increased attention from users, in contrast to a text-based conversational agent (Appel et al., 2012).	↑ Attractiveness
Visual representation	Research indicates that the visual appearance of ECAs significantly affects user perceptions and emotional responses. An anthropomorphized Muppet, which did not resemble a human or an existing animal, elicited the most negative emotions and led users to maintain the greatest interpersonal distance, suggesting discomfort with its unfamiliar design (Bailey and Schloss, 2024). In contrast, an ECA dressed casually in a blue t-shirt, green shorts, and multicolored shoes was perceived as more attractive, indicating that familiar and approachable appearances positively impact user perception (Bailey and Schloss, 2024). Similarly, another study found that users preferred an ECA with supermodel-like features, such as idealized facial traits, symmetry, and a polished appearance, over an ECA with more average and relatable physical traits, which was rated less attractive (Hao et al., 2024). These findings highlight that users tend to favor ECAs with visually appealing, idealized aesthetics. Moreover, ECAs that appeared more realistic were perceived as more conscious and alive compared to cartoon-like ECAs, further emphasizing the impact of realism on user engagement and perception (Volante et al., 2016).	↑ Anthropomorphism ↑ Uncanniness
Customization	Approximately 75% of users valued the ability to customize the ECA’s name, race, and gender, including the option for non-binary identities.	↑ Attractiveness
Data privacy	More than half of the users expressed privacy concerns because ECA frequently requested access to social networks and permission for video calls. Many participants were worried about data storage and manipulation, leading them to deny such requests.	↑ Uncanniness
Gender	Among users, 78% of male participants preferred creating a female ECA, while female users showed more diverse preferences: 59% created a female ECA, 37% chose a male ECA, and 4% opted for a non-binary ECA.	↑ Attractiveness
Communication style	Research shows that ECAs designed to engage emotionally or socially tend to evoke more positive responses from users. For instance, an ECA that used humor, making witty remarks and playful comments about healthy eating, was rated as more attractive and improved users’ moods compared to a non-humorous ECA that delivered directly the factual information (Buttussi and Chittaro, 2019; Hao et al., 2024). Similarly, ECAs that expressed emotions were perceived as significantly more anthropomorphic and emotionally intelligent than those that remained emotionally neutral. Positive messages, characterized by more words, fewer negative terms, and increased use of exclamation marks (e.g., “I’m happy to help!”), further enhanced user perceptions of attractiveness and emotional intelligence (Ham et al., 2024; Ter Stal et al., 2021). Moreover, an ECA using empathic, supportive comments like “You did a good job! Please relax a bit. Then let us continue,” was perceived as more enthusiastic and engaging compared to a task-oriented ECA that simply delivered instructions such as “Next question” (Min et al., 2024). Similarly, a social-oriented ECA, which incorporated personal statements such as “Hello,” and “Have a nice day,” was rated more attractive than a task-oriented ECA that used a more straightforward communication style (van Pinxteren et al., 2023). Interestingly, an ECA that conveyed happiness through captions and emojis was found to enhance perceived emotional intelligence more than the same one expressing emotions like lust, love, or sadness (Ham et al., 2024). Together, these findings suggest that ECAs fostering emotional or social engagement, particularly through humor, positive messaging, and empathetic communication, are consistently rated more attractive, emotionally intelligent, and engaging by users.	↑ Attractiveness ↑ Anthropomorphism ↑ Uncanniness
Facial expressions	Users reported feeling less comfortable and found ECAs with more facial expressions to be less natural (Conrad et al., 2015). However, an ECA displaying emotions such as happiness, warmth, and empathy was perceived as more likeable and caring compared to an unemotional ECA, though there was no significant difference in perceived trustworthiness or intelligence between emotional and neutral-faced ECAs (Creed and Beale, 2012). An ECA that mimicked the user’s head and torso movements with a delay of 1–3 s was not rated as more attractive (Hale and Hamilton, 2016). Trust in ECAs varied depending on the emotions they expressed. Users generally lacked trust in an ECA displaying disgust, while an ECA expressing happiness made them feel more at ease and increased their willingness to cooperate (Luo et al., 2023). Interestingly, reactions to happy ECAs were mixed—while over half of participants viewed them as friendlier, more cooperative, and cordial, others found them insincere, describing them as having a “fake smile” or being “too enthusiastic to be trustworthy.” Most participants were distrustful of ECAs showing negative expressions, often characterizing them as “angry,” “uncooperative,” or “aggressive,” although a small group associated these negative expressions with professionalism and seriousness, finding them more trustworthy. Smiling ECAs were generally perceived as warmer and more cheerful compared to non-smiling ECAs (Min et al., 2024). Lastly, while there were no subjective differences in user perceptions between a flat-faced ECA, an ECA that mimicked user expressions, and one with emotionally adaptable expressions, participants spent more time interacting with the ECA displaying emotionally adaptable facial expressions (Wang et al., 2021).	? Attractiveness ↑ Uncanniness ↑ Anthropomorphism
Non-verbal features	Users with an ECA with many non-verbal features reported greater enjoyment and rated the ECA as more autonomous, personal, less distant, and more sensitive compared to those interacting with less non-verbal features (Conrad et al., 2015). Non-verbal features included adaptive behaviors such as waiting for eye contact, pausing if the respondent stopped looking, offering help when needed, and addressing interruptions immediately (Conrad et al., 2015).	↑ Attractiveness
Voice	Approximately, 68% of users in the No-Emotion condition criticized the ECA’s “irritating, bland voice tone” and described it as “sounding patronizing.”	↑ Uncanniness
Agency	An ECA was perceived as less anthropomorphic than a human partner when collaborating on a shared task (Falcone et al., 2022). However, when users were informed that the ECA’s interactions were controlled by a real human, they found the ECA to be more realistic, humanlike, and helpful compared to when they believed it was controlled by a computer algorithm (Neumann et al., 2023). Interestingly, ECAs with self-oriented mentalization abilities—such as expressing their own feelings (e.g., feeling their own hunger) - evoked stronger feelings of uncanniness compared to ECAs with other-oriented mentalization abilities, which focused on understanding the user’s feelings (e.g., recognizing the user’s hunger) (Yin et al., 2021). The self-oriented focus of these ECAs can appear unsettling and contribute to the UVE (Yin et al., 2021).	? Anthropomorphism ↑ Uncanniness
Ethnical similarity	The ECA that shared ethnic similarity with the user was not perceived as more attractive (Hale and Hamilton, 2016).	? Attractiveness
Personality	The extroverted ECA, which initiated conversations with users, was rated as more natural and agreeable compared to the introverted ECA (Prendinger and Ishizuka, 2001). Additionally, users reported a stronger sense of social presence when interacting with the extroverted ECA. This was likely influenced by the extroverted ECA maintaining direct eye contact 90% of the time, while the introverted ECA only made eye contact 30% of the time during the interaction (Saad and Choura, 2022).	↑ Attractiveness ↑ Anthropomorphism
Emotion recognition	Users initiated more interactions with an ECA that provided emotion recognition, but it was not rated as more attractive (Schouten et al., 2017).	? Attractiveness
Emotional congruence	In a game scenario where the ECA was programmed to lose and the participants to win, a notable increase in participants’ stress levels was observed when the ECA exhibited expressions of joy rather than sadness (Prendinger et al., 2006).	↑ Uncanniness

#### Examination of embodied conversational agent features related to the UVE outcomes

In our review, we observed that ECAs studied were female, half-body, and dynamic in approximately one-third of the cases. Full-body ECAs generally elicited higher levels of anthropomorphism but were also more prone to triggering the uncanniness feelings, especially when their motion was dynamic (Buttussi and Chittaro, 2019). Conversely, half-body and face-only ECAs, while less anthropomorphic, received lower uncanniness ratings (Conrad et al., 2015). The most studies included in the systematic review (18 papers, 62%) examined how various features of the ECA influence user perceptions, such as physical features, facial expressions, communication style, and personality factors (Table 3). Among these features, the facial expressions (6 papers, 20%), and communication style of the ECA were the most extensively explored (6 papers, 20%), but the findings were mixed. A customizable ECA proved to be more attractive, offering users the flexibility to change its gender, race, and name (Belda-Medina and Calvo-Ferrer, 2022). The customisation feature seems to be more important to female users. Overall, users of both genders showed a preference for a female ECA, though choices also included male and non-binary ECAs. Interestingly, an ECA that resembled the user ethnically did not necessarily enhance attractiveness. Furthermore, one study with a good methodological quality found that an ECA with a celebrity appearance in a highly anthropomorphic condition was perceived as less uncanny than the same celebrity represented with a cartoonish appearance (Song and Shin, 2022). When familiar faces are presented in low-anthropomorphism styles, they may trigger stronger feelings of uncanniness, likely because users expect a more realistic physical features when the ECA is based on a real person.

While some research indicated that ECAs with a range of facial expressions were considered more attractive than those without any expressions, the results were not uniform. Eye gaze alone cannot induce uncanniness (Zheleva et al., 2023), probably because more facial features are required in order to induce uncanniness. ECAs with facial expressions were rated higher in terms of perceived compassion and intelligence, and they seemed to encourage more interactions initiated by users (Luo et al., 2023; Min et al., 2024). Moreover, participants spent more time interacting with the ECA displaying emotionally adaptable facial expressions (Wang et al., 2021). However, inconsistencies arose within the same studies. For instance, users did not consistently rate the ECA with facial expressions as more attractive (Conrad et al., 2015; Hale and Hamilton, 2016). Furthermore, the presence of facial expressions in the ECA did not necessarily lead to perceptions of increased friendliness when compared to an ECA without facial expressions (Creed and Beale, 2012). Interestingly a slightly higher number of studies concentrated on positive emotions such as happiness and joy, but some studies examined the effect of negative emotions such as disgust. Users generally perceived positive facial expressions as being more friendly and trustworthy. In contrast, negative expressions were generally associated with lower trust, with users often describing such ECAs as unfriendly or unapproachable (Luo et al., 2023). However, around 60% of users said ECAs with positive expressions looked the friendliest, whereas only 3% did so for those displaying disgust. Interestingly, a small number of users perceived the disgusted ECA as more professional (Luo et al., 2023). Fearful expressions were found to increase fear among users, especially those who had received prior safety training (Buttussi and Chittaro, 2019). These findings suggest that emotional expressions influence user perceptions and emotional responses, but their impact may depend on context, user expectations, and task relevance, with no universally optimal emotional strategy.

In addition to non-verbal cues, the systematic review also explored the role of verbal communication in ECAs. The findings suggest that ECAs with enhanced verbal communication skills are often perceived as more user-like and attractive. Specifically, an ECA expressing joyful messages is regarded as more attractive and helpful compared to an ECA neutral messages (Buttussi and Chittaro, 2019; Ham et al., 2024; Hao et al., 2024). ECAs that used personal greetings like ‘Hello,’ and “Have a nice day,” were rated as more attractive than those with a more straightforward communication style (van Pinxteren et al., 2023). Notably, ECAs that conveyed happiness through captions and emojis were perceived as having greater emotional intelligence than those expressing other emotions like lust or sadness (Ham et al., 2024). Joyful messages were characterized by the use of more words, positive affect terms, and expressive punctuation, like exclamation marks (e.g., “I am happy to help!”). Humor such as amusing stories can enhance the perceived attractiveness of an ECA (Hao et al., 2024). Furthermore, an ECA that engages in friendly communication, evidenced by initiating conversations and employing phrases such as “I’m sorry,” was typically perceived as more attractive. This perception remains consistent regardless of the ECA being represented as male, which was a less-used gender representation in the present review (Prendinger and Ishizuka, 2001). Beyond the quality of information received from ECAs, socio-emotional capabilities were also valued. The ECA could recognize and reflect the user’s emotional state with messages like “It seems you are facing some challenges” and put these feelings into a broader context by saying: “Many people may encounter these difficulties” (Schouten et al., 2017). Additionally, the ECA’s social skill of not interrupting the user during a conversation can make users initiate more interactions (Schouten et al., 2017). Expressions of verbal encouragement, blending affirmation and motivational feedback, such as “Keep going, you are doing well!” can help alleviate user stress (Neumann et al., 2023). This effect supports the extension of the Buffering Stress Theory, traditionally applied to interpersonal relationships, to interactions between humans and ECAs.

Few ECAs were not limited to scripted interactions but could actively recognize and adapt to the user’s emotional states, analyzed through valence-arousal mapping of emotions (Prendinger et al., 2006). Emotional states of the user were detected based on physiological data, including skin conductance (i.e., measured via electrodes on the index and small fingers of the dominant hand of the user) and facial electromyography (i.e., with sensors placed on the use’s left cheek). This input allowed the ECA to classify emotional states based on valence (positive–negative) and arousal (low or high). For example, the emotion “relaxed” has a positive valence and a low arousal (Lang, 1995). Another similar framework, PAD, classifies the emotional states based on three dimensions: Pleasure (vs. displeasure), Arousal (vs. sleepiness), and Dominance (vs. submissiveness) (Becker-Asano, 2008; Kshirsagar, 2002; Russell and Mehrabian, 1977). This framework allowed the ECA to express 18 emotional states including: hopeful, peaceful, bored, annoyed, neutral, depressed, sad, happy, surprised, anxious, angry, overwhelmed, afraid (Sajjadi et al., 2019). These emotions were used to simulate personality traits based on Big Five model (Digman, 1997). For instance, the extraverted ECA expressed emotions that were high in dominance. Such an ECA was sociable, assertive, and maintained direct eye contact for 90% of the interaction time with the user (Sajjadi et al., 2019). In another study, an extroverted ECA, which initiated communication and showed positive emotions such as gratitude, was perceived by the users as attractive (Hao et al., 2024). However, an extroverted ECA can also show anger, manifested through mild frown eyebrows, direct eye contact, shoulders up and sideway posture (Sajjadi et al., 2019). In contrast, an introverted ECA was characterized by emotions low in dominance. Such an ECA was more submissive, showed lower assertiveness, and expressed more negative valanced emotions such as sad, overwhelmed and afraid, with the latter conveyed through slightly raised eyebrows, avoided eye contact, dropped shoulders, and a hand placed on legs (Sajjadi et al., 2019). An introverted ECA maintained eye gaze only 30% of the interaction time with the user, compared to 60% during a neutral emotional state, and it was perceived as more unsettling. While extraversion has received more attention in ECA design, other personality traits have been less frequently explored. For example, only one study focused on the agreeableness personality factor, where an ECA perceived as helpful and forgiving was also considered more attractive by users (Prendinger and Ishizuka, 2001). However, traits such as neuroticism, openness to experience, and conscientiousness were largely neglected in user-ECAs interactions.

Furthermore, most of the included studies have focused on the perceived attractiveness of the ECA, probably because this dimension closely mirrors patterns observed in human social interactions. In contrast, the experience of uncanniness is less well understood, particularly because we lack well-established theories of the UVE in interpersonal contexts. As a result, researchers are still working to interpret and reconcile the often inconsistent findings related to this phenomenon. In the reviewed studies, the ECA perceived as most uncanny was also rated highest in anthropomorphism, specifically in terms of both physical and mental features. One plausible explanation is that users tend to expect ECAs to behave in a mechanical, task-oriented manner. When an ECA displays a high degree of autonomy, such as planning, expressing emotions, or demonstrating independent reasoning, this may conflict with users’ expectations and elicit discomfort (Yin et al., 2021). To counteract this effect, some researchers propose designing ECAs to appear more dependent on human guidance and less capable of fully autonomous behavior. However, the relationship between anthropomorphism and user perception is not straightforward. While greater anthropomorphism may increase the risk of uncanniness, it simultaneously raises users’ emotional expectations. For instance, ECAs with highly human-like appearances are often expected to be more emotionally attuned and responsive (Zhang et al., 2024). When such ECAs successfully express empathy or concern, they tend to be perceived as more attractive. This alignment between anthropomorphic appearance and high emotional features can reduce feelings of uncanniness. Conversely, when emotionally expressive expectations are unmet, users may react more critically, especially toward ECAs that appear highly human.

#### Summary of the main findings

**Figure 2:**
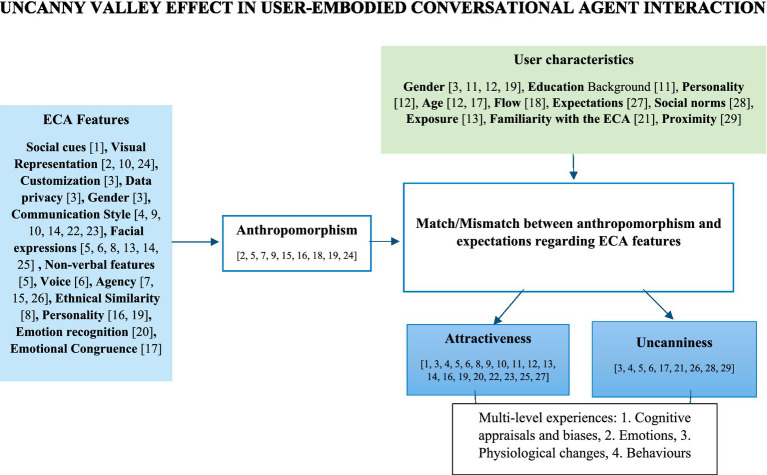
Proposal for an integrative model regarding factors contributing to the UVE in user-ECAs interactions.

In Figure 2, we present a synthesized overview of the evidence related to factors associated with the UVE outcomes in the studies reviewed. The figure is partially data-driven, based on the findings from the included studies that examined either ECA features or user characteristics. The summary figure draws inspiration from previous work (Loveys et al., 2020a). Our analysis revealed significant relationships between several *user characteristics*, such as gender and age, and perceived attractiveness of the ECA. We found a significant association between UVE outcomes and various *ECA features*, including non-verbal features, customization options, humor, friendliness, ECA familiarity. However, we did not find any significant associations between ethnical similarity of the ECA and UVE outcomes. Importantly, the evidence displayed inconsistencies, particularly regarding the relationship between facial expressions exhibited by ECAs and UVE outcomes. Figure 2 not only summarizes the most important results in the included studies but also tries to extend them based on previous theories that can leverage our understanding of user-ECA-interaction.

#### Proposal for a new integrative framework of the UVE in user-ECA interaction

This framework builds on the findings of the included studies in the present systematic review (Table 3), which informed our recommendations for reducing the UVE in user-ECA interaction (Table 4). To situate these results within a broader theoretical context, we draw on three key models: Cognitive Violation Theory (CEVT) (Burgoon and Hale, 1988; Burgoon and Walther, 1990; Kätsyri et al., 2015), the ABC model from the Rational Emotive Behavior Therapy (REBT) (Ellis et al., 2011; Turner, 2016) and the concept of the Uncanny Valley of Mind (UVM) (Gray and Wegner, 2012; Stein and Ohler, 2017). CEVT highlights how mismatches between user expectations and ECA behavior influence user perceptions, while the ABC model from REBT explains how such violations trigger emotional, physiological and behavioral responses from the user. UVM emphasizes the role of mind perception in human ECA-interaction and analyzes the interaction beyond the mere appearance of ECA.

**Table 4:** Checklist for avoiding the Uncanny Valley Effect in ECAs.

No.	Suggestion	Check
1.	**Optimize physical appearance** Design ECAs with polished and aesthetically idealized features, such as symmetrical facial traits, to enhance user perceptions of attractiveness. Users are more likely to respond positively to ECAs with refined, realistic appearances over those with overly simplistic or cartoonish designs. Striking a balance between realism and appeal can reduce feelings of uncanniness while fostering a more engaging interaction.	□
2.	**Optimize Non-verbal Features** Focus on incorporating human-like non-verbal behaviors such as gaze direction, body movements, and response timing. ECAs should utilize natural pauses (e.g., waiting for eye contact from the user), which promote social presence. Include gestures like blinking, head nodding, and gaze shifts, which signal attentiveness and engagement. Studies show that these behaviors significantly enhance attractiveness, thus reducing the sensation of uncanniness. Avoid stiff, robotic movements, as constant staring or mechanical gestures increase discomfort and feelings of uncanniness.	□
3.	**Include Customization Options** Provide users with options to personalize the ECA’s features (e.g., name, race, gender, and even voice). Customization allows for more user familiarity and relatability, increasing attractiveness and reducing discomfort. Research indicates that female users, in particular, report higher satisfaction when they can customize the ECA’s visual appearance. Offering diverse design options, including male, female, and non-binary ECAs, helps meet individual preferences, further enhancing positive user engagement.	□
4.	**Improve Communication Style** ECAs should avoid robotic or overly formal language. Instead, they should use natural, emotionally intelligent communication, incorporating supportive phrases like “You’re doing great!” or “Take your time.” Additionally, the tone of voice is important. Monotone voices are often perceived as patronizing and can increase uncanniness. To mitigate this, ECAs should utilize subtle intonations, appropriate pauses, and a varied vocal range to make their speech more expressive and human-like, fostering greater user comfort and engagement.	□
5.	**Avoid Extreme Emotional Incongruence with the Context of Interaction** Ensure that the ECA’s emotional expressions align with the context of the interaction. Incongruent emotions, such as expressing extreme joy when the user is stressed, can amplify the uncanny effect. ECAs should be able to subtly shift their emotional expressions based on the user’s emotional state to maintain alignment with the situation and reduce discomfort.	□
6.	**Leverage Familiar Interaction Scenarios** Repeated exposure to ECAs can lead to greater acceptance and comfort over time. Gradual exposure to ECAs in familiar contexts can help users build trust and reduce the UVE. Incorporating familiar settings and interaction scenarios allows users to acclimate more easily, decreasing the likelihood of negative responses.	□
7.	**Prioritize Emotional Intelligence and Empathy** ECAs should display emotionally adaptive and consistent expressions. Subtle emotions such as soft smiles, blinking, and empathetic gazes foster trust and relatability. Avoid exaggerated or erratic emotional displays, as emotions like disgust or anger were found to elicit negative reactions. ECAs that show a balanced range of emotional expressions are perceived as more natural and approachable.	□
8.	**Incorporate Social and Cultural Norms** ECAs that reflect or are sensitive to the user’s cultural background can enhance feelings of familiarity and trust. Consider incorporating cultural cues, such as language, accent, or behavior, that resonate with the user’s identity. ECAs that are perceived as culturally aligned are less likely to invoke the Uncanny Valley Effect.	□
9.	**Expectations and Anthropomorphism** Users generally have higher expectations for highly anthropomorphic ECAs, and when these expectations are not met, the sense of uncanniness can intensify. Therefore, it’s crucial to evaluate user expectations early in the interaction process. ECAs must effectively manage these expectations by demonstrating understanding and empathy toward the user’s concerns. The closer an ECA aligns with user expectations in terms of emotional responsiveness, the less likely users are to experience discomfort or feelings of uncanniness. Meeting these expectations enhances user trust and engagement, helping the ECA to appear more natural and relatable.	□

To better understand the UVE in user-ECAs interaction, the present framework goes beyond an exclusive focus on the ECA’s features and considers the user’s experience as a central component. We propose a model in which the UVE emerges from the dynamic interplay between ECA features and individual user characteristics based on the results from our included studies on users factors and ECA features (see Figure 2). The interaction begins with a trigger, which is a specific feature of the ECA (e.g., clothing, facial expression, gesture or communication style) as depicted in the studies included in the systematic review (Bailey and Schloss, 2024; Hao et al., 2024; Volante et al., 2016). This trigger activates cognitive appraisals in the user, such as judgments about the ECA’s degree of anthropomorphism: “This ECA is like a human being.” Before the interaction even starts, however, users bring their own factors into the experience. Characteristics such as gender, age, previous experiences, and personality traits were explored in the included studies (Belda-Medina and Calvo-Ferrer, 2022; Lahav et al., 2020; Lisetti et al., 2004; Luo et al., 2023; Prendinger et al., 2006; Sajjadi et al., 2019) shape their expectations and influence how they interpret the ECA’s features. Following the initial appraisal, the user evaluates whether the ECA matches or mismatches their expectations. Given the human brain’s predictive nature, a match typically leads to attractiveness. However, individual traits can moderate this process. Users high in openness to experience may perceive an unexpected or mismatching ECA as both attractive (Lisetti et al., 2004) and uncanny, driven by curiosity (Zibrek et al., 2018). In contrast, users with high trait anxiety may respond to mismatching ECAs with uncanniness, potentially perceiving them as a threat.

It is essential to redefine the outcomes of the UVE across four key levels, grounded in validated theories of Psychology (Ellis et al., 2011). The first and most critical level is cognitive, or how the user thinks about the ECAs. For example, it is important to know whether they perceive it as competent or incompetent, friendly or unfriendly. These cognitive appraisals shape the second level, which is emotional. Here, we assess the user’s emotional response to the ECA, such as feeling relaxed, uncomfortable, surprised, curious, disgusted, or anxious. The third level involves physiological responses, such as changes in skin conductance or heart rate, which indicate levels of stress or relaxation. Finally, the fourth level is behavioral, where we examine how often the user maintains or even initiates the interaction with the ECA. Evaluating outcomes across all four levels is essential for a comprehensive understanding of user experience with ECAs.

In designing and evaluating human-ECA interactions, it is important to consider that users may naturally perceive and respond to ECAs as if they were human partners (Scheele et al., 2015). This opens the door for social cognition to play a role in these interactions with its well-known cognitive biases. One example is the hostile attribution bias, which is the tendency to see unclear behavior as hostile (Birch et al., 2025). In the context of ECAs, an ambiguous response might be misinterpreted negatively, as rude or even aggressive. Another example, anchoring bias causes users to rely too heavily on their first impression of the ECA, even if later behavior is different (Qi, 2024). Lastly, negativity bias means that users give more weight to negative experiences than to positive ones (Vaish et al., 2008). Thus, one awkward moment with the ECA can ruin the entire interaction. These well-known cognitive tendencies come from research on how humans relate to other people. A well-designed ECA should minimize ambiguity, promote trust from the start, and recover gracefully from small mistakes.

## Discussion

This study aimed to provide the first comprehensive systematic review to investigate the UVE in user-ECA interaction, with a specific focus on three outcomes: (a) anthropomorphism (9 papers, 31%), (b) attractiveness (29 studies, 65%), (c) uncanniness (9 papers, 31%), with some studies looking simultaneously at more than one outcome (7 papers, 24%). Our review followed the PRISMA guidelines, and we meticulously examined 29 published studies to identify potential three key aspects: (1) user characteristics, and (2) ECA features related to the UVE outcomes. Below, we delve into the key findings derived from our work.

### To what extent is the UVE present in user interactions with ECAs?

It is essential to assess the UVE through a comprehensive combination of attractiveness, uncanniness, and anthropomorphism. Focusing solely on attractiveness can offer useful insights into ECA design, but it overlooks the full range of potential discomfort that users might experience. A comprehensive evaluation across all three variables is crucial to better understand and mitigate the UVE.

Approximately one-third of the studies in this systematic review specifically focused on UVE as a primary goal, and these studies successfully confirmed its presence. However, many of the remaining studies only explored UVE-related variables as secondary objectives, with a predominant emphasis on the attractiveness of the ECAs. These studies did not directly examine the transition between attractiveness and uncanniness, which is critical to fully understanding the UVE.

In the studies that confirmed the UVE, fewer than half utilized standardized measurement tools, such as the Godspeed Indices (Tobis et al., 2023) or the Ho and MacDorman (2017). Instead, many opted to create custom measurement items, which introduced variability in how the UVE was assessed. Despite this, nearly all studies employed randomization, whether through within-subject or between-subject designs, underscoring the critical importance of randomization in reducing potential biases. In terms of methodological quality, 62% of the included studies were rated as having moderate quality, while the rest were rated as weak, making them susceptible to bias. Future research should address these biases and systematically study the UVE to minimize risks and improve the robustness of findings.

### How can we avoid the Uncanny Valley Effect?

#### User profile characteristics

Several studies demonstrated that female users consistently rated ECAs as more attractive and reported lower levels of uncanniness compared to male users (Belda-Medina and Calvo-Ferrer, 2022; Lahav et al., 2020; Lisetti et al., 2004; Sajjadi et al., 2019). This may be attributed to higher levels of empathy in female users, which can positively influence their perception of ECAs. Men, on the other hand, often exhibit greater skepticism toward human-like ECAs, finding it more difficult to fully accept the agent’s anthropomorphic qualities. However, these findings should be interpreted with caution because some studies suggest that females are more likely to find ECAs attractive (Foster, 2007), while others argue the opposite, claiming males are more inclined to do so (Kuo et al., 2009). In terms of uncanniness, research suggests that female users may be more susceptible to feelings of uncanniness (MacDorman and Entezari, 2015), possibly due to a greater sensitivity to disgust compared to men (Tybur et al., 2011).

Additionally, younger participants, particularly those experiencing flow during the engagement, tended to perceive ECAs more favorably, with higher ratings of attractiveness and fewer reports of discomfort (Lisetti et al., 2004; Prendinger et al., 2006), In contrast, older users tend to be more sensitive to the UVE, likely due to a heightened awareness of the agent’s human-like but imperfect features. Contrary to our findings on human-ECA interactions, research on human-robot interaction shows that users aged 18–59 are more likely to find robots uncanny compared to older users (60–87 years) (Tu et al., 2020), which could be linked to enhanced emotion regulation skills in older individuals (Carstensen, 1995). Moreover, previous research shows that young children (ages 3–5) may not experience the UVE in the same way as other age groups (Tu et al., 2020; Brink et al., 2019).

Even the educational background makes a difference in which UVE studies should be mindful when pulling together different domains. While Social Sciences students may be less familiar with cutting-edge technical advancements, they may exhibit greater openness to engaging with ECAs. When asked whether they would apply career recommendations from their interactions with ECAs in their daily life, social sciences and humanities users were notably more likely to respond positively compared to users from exact sciences, medicine, and engineering. Based on this review, the ideal user profile for effective interaction with ECAs appears to be young, preferably Gen Z, females, preferably with a background in Social Sciences and a high level of openness to experience. These users generally show greater openness and comfort with ECAs, demonstrating a higher tolerance for minor imperfections in the agent’s behavior. Their social awareness enables smoother engagement with ECAs, significantly reducing the likelihood of experiencing the UVE.

However, these results should be interpreted with caution, because we had limited demographic categories to compare, especially when we investigated the educational background, where we found differences in users from just two educational areas: Social Sciences and Exact Sciences.

#### ECA features

In our review, we observed that ECAs studied were female, half-body, and dynamic in approximately one-third of the cases. Full-body ECAs generally elicited higher levels of anthropomorphism but were also more prone to triggering the uncanniness feelings, especially when their motion was dynamic (Buttussi and Chittaro, 2019). Conversely, half-body and face-only ECAs, while less anthropomorphic, received lower uncanniness ratings (Conrad et al., 2015). Interestingly, studies using scripted ECAs confirmed the UVE in only 2 out of 9 cases (Conrad et al., 2015; Zheleva et al., 2023), suggesting that limited interactivity may reduce the likelihood of eliciting uncanniness responses.

Studies indicate that ECAs designed with complex communication features often yield more positive responses. For example, an ECA incorporating humor and playful comments about healthy eating was rated more favorably and improved users’ moods compared to a non-humorous counterpart delivering factual information; (Buttussi and Chittaro, 2019; Hao et al., 2024). ECAs that express emotions are perceived as more anthropomorphic and emotionally intelligent than those that maintain emotional neutrality. Positive messaging, characterized by a higher word count, fewer negative terms, and greater use of exclamation marks (e.g., “I’m happy to help!”), further enhances perceptions of attractiveness and emotional intelligence (Ham et al., 2024; Ter Stal et al., 2021). ECAs providing empathic and supportive feedback, such as “You did a good job! Please relax a bit. Then let us continue,” were seen as more engaging and enthusiastic compared to those that were purely task-oriented (Min et al., 2024). Users were nearly twice as likely to show acknowledgement responses, such as verbal affirmations like “yes” or “aha,” when the ECA displayed more facial expressions, including movements of the head, eyes, mouth, gaze direction, and subtle blinking patterns (Conrad et al., 2015). Previous literature showed that while expressing empathy can make ECAs more appealing (Parmar et al., 2022), it can simultaneously increase their uncanniness (Gray and Wegner, 2012; Stein and Ohler, 2017). This uncanniness is frequently intensified when ECAs exhibit anthropomorphic appearance, emotions or consciousness (Diel et al., 2021; Ho et al., 2008). Assigning mental abilities to ECAs, such as emotions or decision-making, exacerbates the risk of the UVE, as users tend to become less willing to interact with them (Yin et al., 2021). Adding to the complexity, some studies suggest that uncanny reactions are stronger when ECAs display basic sensations like hunger or pain (Gray and Wegner, 2012), while others argue that the effect is more pronounced with complex mental traits such as memory and moral judgment (Lu, 2021).

The non-verbal behaviors of ECAs play a crucial role in fostering user engagement. However, their timing is essential; if poorly synchronized or overly frequent, these behaviors may appear unnatural and potentially elicit feelings of uncanniness (Conrad et al., 2015). Therefore, increased engagement does not necessarily correlate with increased attractiveness or user comfort. Reactions to happy facial expressions of the ECAs were mixed. While many participants viewed them as friendlier and more cooperative; some perceived them as insincere or overly enthusiastic. On the other hand, negative expressions often led to perceptions of aggression or uncooperativeness, though a few users associated them with professionalism and seriousness (Luo et al., 2023). Smiling ECAs were generally rated as warmer and more cheerful than those without smiles (Min et al., 2024). Furthermore, users interacting with ECAs featuring rich non-verbal behaviors, such as responding to eye contact, pausing when users stopped looking, and addressing interruptions, reported higher enjoyment and rated these ECAs as more autonomous and natural compared to those with fewer non-verbal features (Conrad et al., 2015). An extroverted ECA, which actively initiated conversations and maintained eye contact 90% of the time, was perceived as more natural and engaging than an introverted ECA that made eye contact only 30% of the time (Prendinger and Ishizuka, 2001; Saad and Choura, 2022). However, results must be interpreted with caution, because these studies were rated as having either weak (Conrad et al., 2015; Luo et al., 2023; Min et al., 2024) or moderate overall methodological quality (Prendinger and Ishizuka, 2001; Saad and Choura, 2022).

The findings across the included studies suggest that mitigating the UVE requires ECAs to incorporate expressions of happiness, show concern, and exhibit social behaviors. Engaging in active listening, providing encouragement, and aligning emotional expressions with user expectations and context are crucial for creating a natural and satisfying experience. Customizing emotional displays based on user preferences, supported by insights from relevant datasets, such as the MuFaSAA Dataset, can significantly improve interaction quality and user satisfaction. Understanding user expectations, affective, cognitive, and behavioral, will aid in designing ECAs that are both effective and engaging. In response to these findings, we have developed a preliminary checklist focused on key design features of ECAs that may reduce the likelihood of UVE. This checklist is intended as a practical starting point for designers and developers working to improve the emotional and social realism of ECAs.

### How can we advance the study of the UVE in user-ECA interactions?

Although the design features of ECAs are undoubtedly important, an exclusive focus on them reveals a significant gap in the literature. Most studies treat users as a homogeneous audience and, as a result, discover inconsistent findings. As previous literature suggests, it is essential to “bring back the human” in human-ECA interaction (Arora et al., 2021) and place a greater emphasis on the subjective experience of the user. The UVE should not be considered a universal experience anymore. As illustrated in our proposed integrative framework (Figure 2), the UVE does not emerge solely from the ECA’s features, but from how these features are cognitively interpreted by the user. Our framework addresses this gap by placing cognitive appraisal at the center of the user-ECA interaction. The UVE is conceptualized here as a product of mismatch detection between the perceived level of anthropomorphism and the user’s expectations. Users do not passively perceive ECAs, but they actively construct meaning based on internal models of social interaction. These cognitive appraisals are influenced not only by the ECA’s features (e.g., physical features, verbal and non-verbal features), but also by the user’s gender, personality, familiarity with ECAs, and other psychological predispositions. Importantly, our model encourages researchers to assess user experience across four levels: (1) cognitive appraisals and potential biases, (2) emotions, (3) physiological arousal, and (4) behaviors. This multi-level structure not only aligns with established cognitive science theories but also provides a more nuanced account of how users respond to ECAs.

Our framework expands prior work by addressing the expectancy violations not only at a perceptual level (i.e., user’s perceptions of the ECA’s appearance) (Kätsyri et al., 2015), but also at a cognitive (i.e., users’ perceptions of the ECA’s mind), emotional (i.e., users’ perceptions of how ECAs express both verbally and non-verbally), and behavioral levels (i.e., how the ECA acts in the interaction). This multi-layer approach builds on evidence of the Uncanny Valley of Mind, where mismatches in the perceived mind of the ECA can produce uncanniness (Gray and Wegner, 2012; Stein and Ohler, 2017). This framework can be particularly useful when users are actively interacting with the ECAs through brief conversations, interviews, training sessions or complex gaming scenarios. When the ECAs violate the user’s expectations regarding how the ECAs should look, think, feel or act, users may experience uncanniness. This is supported by neuroscientific findings suggesting that the UVE may stem from violations of the brain’s predictive models when users interact with anthropomorphic ECAs (Urgen et al., 2018). CEVT has proven relevant in human-robot interaction (Claure et al., 2020), and there are efforts to apply this to human-ECA interactions as well. For instance, positive expectancy violations, where an ECA exceeds user expectations, can enhance satisfaction and perceived connectedness, while negative expectancy violations can lead to disappointment or even unease (Grimes et al., 2021).

Both CEVT and REBT emphasize the central role of cognitive processes of the users. However, REBT extends this perspective by explicitly linking cognitive appraisals to users’ emotional, physiological, and behavioral reactions, offering a comprehensive model for analyzing user experience (Ellis, 2003). Originally developed in a clinical context, REBT has been integrated into ECAs designed for early detection of suicidal ideation, support in depression treatment and promoting positive health behavior change (Burton et al., 2016; Lisetti et al., 2013; Martínez-Miranda et al., 2019). Importantly, REBT assumptions remain relevant even in non-clinical samples of users. Features of the ECA, such as facial expressions or gestures, can act as triggers for the user’s underlying beliefs and expectations. By combining CEVT with REBT, we advocate for a multi-layered understanding of the UVE, one that acknowledges the interplay between cognitive, emotional, physiological, and behavioral responses of the users when interacting with an ECA.

### Limitations

One of the most important limitations of the present systematic review is that the studies we reviewed focused on only one aspect of UVE, specifically the attractiveness of the ECA. Future research should adopt a more holistic approach, examining all three key outcomes, utilizing a variety of data collection methods that span subjective, behavioral, and physiological measurements. Another major limitation of this review is the considerable heterogeneity among the included studies in terms of design, measurement tools, and reported outcomes, which precluded the possibility of conducting a meta-analysis.

Furthermore, the majority of the studies were classified as having “moderate” or “weak” overall methodological quality. As a result, the findings should be interpreted with caution, as the potential limitations in study design and rigor may influence the reliability of the conclusions drawn. This indicates that while these studies present certain strengths, they also exhibit notable limitations. Additionally, while we calculated inter-rater agreement for the quality appraisal of the included studies, we did not conduct inter-rater reliability procedures during the data extraction phase. Data extraction was performed by one author, with ongoing consultation and consensus discussions with a senior co-author. Nevertheless, the absence of independent double coding means that some degree of individual bias cannot be entirely ruled out, despite our best efforts to ensure accuracy and consistency.

Subjective measurement tools must be both valid and reliable to accurately capture user experiences. Yet, few studies have calculated coefficient alpha to ensure internal consistency, and while some relied on pre-validated questionnaires, others developed their own singular items like, “This ECA is attractive.” Such isolated measures risk oversimplifying the complexity of user perceptions, failing to capture the full spectrum of emotional and cognitive reactions. Though some validated scales exist (Bartneck et al., 2009; Ho and MacDorman, 2017), they often employ opposite adjective pairs, which can oversimplify the nuances of user experiences, potentially distorting the full picture of the UVE.

However, a key limitation is that most studies assess UVE primarily through subjective self-reports of attractiveness, anthropomorphism, and uncanniness. Although these perceptions are important, the reliance on subjective measures alone limits our understanding of how UVE might manifest at a deeper, unconscious level. For example, physiological or behavioral indicators such as eye-tracking, heart rate, or skin conductance are often absent, despite evidence that objective measures do not always align with subjective perceptions (Schouten et al., 2017; Wang et al., 2021; Zheleva et al., 2023; Zibrek et al., 2018). Unfortunately, only a minority of studies incorporate these objective measures, leaving a critical gap in the literature. Future research should aim to combine both subjective and objective data to provide a more comprehensive understanding of UVE.

Another concern is the short duration of most user-ECA interactions, typically lasting only a few minutes. While these brief encounters are useful for gauging initial impressions, they do not account for how perceptions might evolve with prolonged or repeated exposure. The lack of longitudinal studies makes it difficult to assess the durability of UVE. For instance, would repeated interactions with an ECA help alleviate the discomfort associated with the Uncanny Valley? Current research does not adequately address this, leaving an important question unanswered. There is evidence that suggests user acceptance of ECAs improves over time (Lisetti et al., 2004). This highlights the need for longer-term investigations into UVE.

Another shortcoming of the present paper concerns the limited attention to advanced affective and personality modeling in the included studies. In more than half of the included studies, the computational architecture of the ECAs was either not clearly described or difficult to extract. Only a few studies clearly stated the PAD framework (Prendinger et al., 2006; Sajjadi et al., 2019), and none employed complex models such as ALMA, which integrates emotion, mood, and personality analyzed through PAD dimensions (Gebhard, 2005). None of the studies presented Affect Control Theory (ACT), which explicitly links the ECA’s emotional expressions to the social context of the interaction (Lively and Heise, 2014; Sandercock et al., 2006). Complex ECAs should rely on Bayesian networks to infer the user’s emotional states and personality, and further, generate adapted behaviors in the ECA through verbal utterances, speech rhythm, pitch, gestures, facial expressions and body language (Breese and Ball, 1998). The architecture of the ECA should allow for dynamic alignment the ECA’s affective state with the user’s emotional profile, a strategy that holds promise in mental health care provided by ECAs (Siemon et al., 2022). Unfortunately, most of the ECAs in the included studies adopted a one-size-fits-all approach rather than tailoring the ECA’s behavior to the individual users. Future ECAs can analyze user-generated test data through chat logs or social media content of the user to infer dominant personality traits of the user and adapt their response accordingly, while respecting the privacy of the users. While ACT has been applied successfully in human-robot interaction, it remains underexplored in ECA research, despite its potential to significantly improve user experience (Corrao et al., 2025). A promising implementation of the ALMA architecture in user-ECA interaction is presented in a recent study (Sonlu et al., 2021). Future studies should benefit from building such comprehensive frameworks to support the development of emotionally intelligent and socially adaptive ECAs.

Furthermore, a key methodological concern in the studies reviewed is the lack of randomization in approximately one-third of the research. This absence increases the risk of participant selection bias and design flaws, potentially compromising the validity of the findings. To strengthen the robustness of future research on the UVE, it is essential to consistently implement randomization to reduce confounding variables and provide more reliable conclusions. Authors must ensure that groups are comparable at baseline in terms of potential confounders, such as race, sex, age, education, and income (Thomas et al., 2004), yet most studies were rated as “weak” in this area.

Additionally, the issue of participant withdrawal further weakened the reliability of many studies. Researchers are expected to report the proportion of participants who completed the study and to explain dropouts, if applicable (Thomas et al., 2004). Unfortunately, most of the reviewed studies failed to adequately address participant withdrawal rates, with many neglecting to mention them altogether. This omission raises concerns about the reliability and generalizability of the findings, as unreported withdrawals could significantly affect the outcome and interpretation of results. Moving forward, careful management of these methodological concerns is critical for producing high-quality, trustworthy research in UVE studies.

Lastly, there is inconsistent reporting on key variables across studies. Several studies fail to provide important details, such as gender distribution, age range of users or even clear descriptions of the ECA features (e.g., “Not Clear” for some characteristics). This lack of transparency hampers the ability to replicate findings and limits understanding of how specific variables influence UVE. Additionally, some studies provide vague or missing information about the duration of user-ECA engagement, complicating efforts to compare results across studies. A more rigorous approach to reporting experimental details is essential to advance the field and allow for more accurate cross-study comparisons.

Such methodological improvements are particularly important given the complexity of UVE. Despite the discomfort or confusion often associated with UVE, there is evidence that people exhibit curiosity toward entities that evoke these uncanny responses. This suggests that, while UVE can create unease, it may also provoke curiosity, potentially encouraging further exploration of ECAs (Bailey and Schloss, 2024; Yin et al., 2021; Zibrek et al., 2018). This is particularly significant in immersive media technologies like VR, where UVE tends to be more pronounced (Bailey and Schloss, 2024). Moreover, the UVE appears to be influenced by ECA’s cognitive abilities. For instance, humanlike ECAs with self-oriented mentalization abilities elicit stronger feelings of dislike compared to those with other-oriented mentalization abilities, adding further complexity to our understanding of UVE’s psychological underpinnings (Yin et al., 2021). However, the results of the last study should be interpreted with caution because it received a weak overall methodological quality.

To sum up, the inclusion of studies with weak or medium methodological quality in this review could affect the replicability of findings. Although most studies employed experimental designs, there was a lack of uniformity in their methodological approaches for reporting results. To enhance research rigor, future studies should consider pre-registering their research protocols. Additionally, our review highlighted a gap in longitudinal research, as no study examined the UVE interactions over time. *Secondly*, the majority of the conclusions drawn in this systematic review are based on findings from a limited subset of studies, as illustrated in Figure 2. Moreover, few of the aforementioned results are derived from user-robot interaction literature (Ham et al., 2024; Lisetti et al., 2004; Yin et al., 2021), which can lead to a lack of standardized information on how to improve interaction with ECAs. Furthermore, the user experience in user-ECAs interaction may differ from user-robot interaction. To validate these important discoveries, it is crucial for future research to replicate these effects. In the following section, we offer guidelines aimed at streamlining the replication efforts for experimental investigations into user-ECA interactions, with a particular emphasis on exploring the UVE.

### Implications

The findings of this systematic review carry significant practical and methodological implications for the are of user-ECAs interactions. Below, we outline recommendations aimed at enhancing the quality of user-ECA interactions, as well as suggestions for refining the methodologies employed in future experimental studies within this domain.

#### Suggestions for enhancing interactions with ECAs

Considering our systematic review’s findings, we propose the following strategies to enhance the perceived attractiveness of the ECAs and reduce the likelihood of eliciting uncanny feelings. More details can be found in the *Checklist for Avoiding the Uncanny Valley Effect in ECAs* (see Table 4), which has been developed based on the findings of the present systematic review. By following our recommendations outlined below, designers and developers can reduce the likelihood of triggering the UVE in users.

ECA should adopt a positive attitude: To enhance their attractiveness, future ECAs should exhibit humor (for example, telling jokes or using a sarcastic tone) and friendliness (such as greeting users at the start of interactions or apologizing when unable to assist). In text-based communications, employing a greater volume of words, minimizing negative affect keywords, and increasing positive affect terms, along with the use of punctuation and exclamation marks, can make interactions more attractive (Ter Stal et al., 2021). For instance, phrases like “I am glad to assist you!” can increase the perceived attractiveness of ECAs (Ter Stal et al., 2021).

To make ECAs more attractive, it is essential to enhance both verbal and non-verbal communication. ECAs should not only provide clarifications during interactions, but also interpret cues from the user’s speech and actions to enrich the conversation (Volante et al., 2016). Demonstrating reflective listening, acknowledging the user’s emotions and providing empathetic responses, like “It seems you are feeling scared.”—helps foster a stronger emotional connection and boost user confidence (Schouten et al., 2017). Furthermore, ECAs need to respond to non-verbal cues effectively, such as showing confusion to dismissive gestures or attentiveness to pointing actions (Wang et al., 2021). Research shows that when ECAs mimic natural facial expressions and head movements, even while listening, user engagement improves, often leading to positive responses like increased smiling (Volante et al., 2016).

However, because not all users react the same way, understanding their expectations is also important. Using resources like the MuFaSAA Dataset (Dennler et al., 2023), which provides insights into user preferences, can help tailor these interactions. Adapting ECA behavior based on individual needs, as seen with the Geminoid HI robot, can lead to more effective and personalized interactions. To ensure ECAs meet user expectations and avoid the UVE, it’s important to use reliable metrics. Tools like the Negative Attitude Toward Robots Scale (NARS), the Robot Anxiety Scale (RAS), and measures such as reaction times during interactions can help developers understand user reactions. Aligning ECA behavior with the results from these metrics can prevent discomfort and increase user satisfaction, helping to ensure a positive experience and mitigate.

Future ECAs should offer extensive customization options, starting with gender preferences to enhance their perceived attractiveness. Our included studies presented mostly female ECAs, probably because previous literature shows a general preference for female ECAs (Kulms et al., 2011). Both male and female users generally showed a preference for female ECAs. However, female users tended to be more open to interacting with male or agender ECAs (Volante et al., 2016). Beyond physical features, future ECAs should not only allow for modifications in physical appearance but also offer personalization of personality traits. These traits can be showcased through their voice, facial expressions, and body movements (Ahmad et al., 2022; Sonlu et al., 2021). Users should be able to choose from a range of traits based on the Big Five Personality Model (McCrae and Costa, 1997). A study highlighted that ECAs exhibiting clear signs of extraversion verbally, with phrases like “Yes, I will purchase the return ticket immediately. Thank you, officer,” and showing happiness nonverbally, were perceived as more attractive by users (Ter Stal et al., 2021). Our systematic review suggests a preference for extraversion in ECAs, yet contrasting studies reveal nuanced findings. Research indicates that extroverted ECAs, characterized by quicker speech and more frequent smiles, were deemed less trustworthy than introverted ones by extroverted users (Liew and Tan, 2016; Loveys et al., 2020b). This highlights the diversity in user preferences regarding ECA personalities, underlining the importance of offering customizable traits to accommodate a wide range of expectations.

To prevent eliciting uncanny feelings, future ECAs should be capable of adjusting their emotional responses based on the social context. For instance, in competitive scenarios where the ECA emerges victorious, it should naturally display pride and happiness, even if it means the user participants lose. Likewise, during collaborative tasks where both the ECA and users succeed, the ECA should similarly exhibit feelings of pride and joy. A study investigating the effect of ECAs displaying incongruent emotions in a competitive gaming setting found that ECAs not showing happiness at their own victories led to perceptions of uncanniness among users (Volante et al., 2016). This underscores the importance of ECAs being emotionally in tune with the context to avoid unsettling reactions.

#### Theoretical implications

The UVE is still in its early stages but represents a promising area of research. One major issue is that the UVE has not been clearly defined, and many studies fail to meet minimal methodological standards. To address this, we provided a clear definition for each variable included in the UVE and expanded its scope to encompass not only the physical but also behavioral and mental features of ECAs. Another critical issue is that many of the studies we reviewed did not prioritize the UVE as a primary objective, leading to a lack of rigor and reproducibility. In response, we offered methodological recommendations drawn from experimental psychology, where higher standards of rigor and replicability are common. These recommendations should guide future research toward more robust, reliable findings in this field.

We strongly recommend that future studies move beyond focusing solely on ECA features and instead analyze the UVE in a way similar to how we assess interactions between humans. A continued focus on ECA features alone risks creating overly universal agents that fail to meet personalized needs, leading to low user engagement and poor usability. Future research should also explore the psychological characteristics of users, such as personality traits and clinical factors like anxiety or depression, which may increase the likelihood of experiencing uncanniness (MacDorman and Entezari, 2015). Conversely, conditions such as autism may reduce this effect (Feng et al., 2018). Additionally, studies should investigate contextual factors, such as optimal interaction durations to prevent cognitive overload, as well as appropriate tasks and environments that enhance user engagement with ECAs.

In human interactions, we analyze and predict others’ emotions based on a combination of sensory inputs, past experiences, and contextual factors (Barrett et al., 2011). This same process occurs when interacting with ECAs. Barrett’s theory of constructed emotion is particularly relevant for understanding the UVE because it highlights how users may perceive ECAs’ emotional expressions differently depending on the context. For instance, the same facial expression of the ECAs may be interpreted as welcoming in one setting but uncanny in another. As Barrett argues, when the context changes, so does the emotional interpretation. Future research on ECAs should consider how varying contexts might influence users’ perceptions of the agent’s emotions, which could either mitigate or exacerbate the UVE. In our framework, we analyzed the UVE by considering not only the user and the ECA, but also the context in which the interaction occurs.

There is a pressing need for more accurate methods of assessing the UVE. Nearly half of the included studies used a maximum of five items to measure the UVE, with many relying on single-item assessments, an approach that should no longer be considered acceptable (Sarstedt and Wilczynski, 2009). More nuanced and comprehensive questions are required, along with verification items and scales that have been thoroughly tested for reliability, such as Cronbach’s alpha. Currently, the most widely used tools are the Godspeed Questionnaire Series (Bartneck et al., 2009; Tobis et al., 2023) and the scale proposed by Ho and MacDorman that primarily rely on semantic differential items (e.g., humanlike-mechanical, friendly-hostile). While these instruments are practical and widely adopted, they raise important concerns. First, they predominantly assess perceptual impressions, with limited sensitivity to affective discomfort, ambivalence, or behavioral intentions to use the ECAs. Second, binary adjective pairs are prone to semantic ambiguity and cognitive noise, especially when the adjectives are polysemous. Additionally, these instruments are vulnerable to social desirability bias. Negatively valenced adjectives such as “awful,” “unpleasant,” or “incompetent” may be perceived as socially inappropriate, leading participants to underreport negative reactions. A particularly critical limitation is the lack of empirically established cut-off scores in the existing instruments. Without defined thresholds, it is not possible to determine with confidence when an ECA enters in the UVE zone. In clinical psychological research, cut-off scores are essential for converting continuous subjective ratings into interpretable categories with clinical relevance. For instance, the Brief Emotional Intelligence Scale (BEIS-10) and the Difficulties in Emotion Regulation Scale (DERS) include empirically derived thresholds to classify individuals along relevant dimensions, enabling more precise interpretation and application (Davies et al., 2011; Gratz and Roemer, 2015). Best practices in psychometric development emphasize a three-phase process: item generation, scale construction, and scale validation (Boateng et al., 2018). UVE research would benefit significantly from adopting such an approach to develop robust, multidimensional tools that measure anthropomorphism, attractiveness, and uncanniness as distinct yet related constructs. Critically, future tools should provide normative cut-offs to indicate mild, moderate, or severe UVE responses. Without such developments, the empirical study of user discomfort and avoidance in human-ECA interactions will continue to lack coherence and predictive power.

Moving forward, future research should aim to develop tools that better capture the emotional and social dimensions of the UVE. In this regard, it is crucial to integrate both quantitative and qualitative feedback, as these subjective methods provide valuable insights, but they are not sufficient on their own (Taschereau-Dumouchel et al., 2022). We must also incorporate behavioral (e.g., eye gaze) and physiological measurements (e.g., skin conductance), as some of the included studies have done (Appel et al., 2012; Bailey and Schloss, 2024; Hale and Hamilton, 2016; Schouten et al., 2017). These additional metrics can offer more precise information, allowing for a deeper understanding of the UVE. With these initiatives, UVE research can evolve toward a more comprehensive and accurate approach.

#### Methodological recommendations

Based on our quality assessment, which revealed that most studies were of weak to moderate quality, we emphasize the critical need for methodological improvements in future research. To address this, we propose recommendations aligned with the CONSORT—Consolidated Standards of Reporting Trials (Baker et al., 2010; Eysenbach et al., 2011): CONSORT describes how the interaction with an ECA should be reported. It offers a clear checklist that can be used for randomized controlled trials (RCT), and also non-RCT evaluation reports (Eysenbach et al., 2011):

- Future research should emphasize the importance of defining clear objectives and specific aims. Most of the reviewed studies employed a hypothesis-driven analysis and selected variables based on theoretical considerations. Upcoming studies must be grounded in solid scientific documentation, featuring well-defined aims and articulated hypotheses (Baker et al., 2010; Hariton and Locascio, 2018).
- Future studies should specify eligibility criteria and how the sample size was calculated. Most of the studies received a moderate quality rating for the selection of participants. Future work needs to detail specific inclusion criteria for participants, such as age limits or required levels of technology proficiency, to ensure the replicability of studies. Furthermore, future studies should use specialized software such as the G-Power program for calculating sample sizes and statistical power across a range of analyses like F, t, χ^2^, and Z, (Faul et al., 2007). Employing such precise estimations for required sample sizes will facilitate evidence-based decision-making and judgments in the study designs (Kang, 2021).
- Future studies should provide information regarding participant withdrawal. A substantial number of the studies reviewed were assessed as weak in quality concerning participant withdrawal. These studies often lacked detailed information on both the numbers and reasons for participant withdrawals (Armijo-Olivo et al., 2014; Thomas et al., 2004). Future research should include comprehensive data on participant withdrawal rates to enhance study transparency. This includes documenting the percentage of participants who remain in the study until the final data collection point and providing insights into study completion rates and potential biases resulting from attrition.
- Future research should thoroughly address the issue of confounding variables, as most reviewed studies were rated as weak in controlling for these factors. Notably, only a few studies assessed demographic variables before randomization. Future studies should evaluate certain characteristics of the participants before randomization to confirm that variations between groups occur from the interaction with the ECA and not from baseline differences between groups, which might potentially influence the results (Twisk et al., 2018; Roberts and Torgerson, 1999). Examples of potential confounders include race, sex, age, income, and pre-intervention scores on outcome measures. Such careful examination is necessary to ensure that observed effects, such as the perceived attractiveness of one ECA over another, are not exaggerated by uncontrolled variables. To aid in clarity and transparency, it is advisable to present a table summarizing the baseline demographic characteristics (e.g., occupation, education, etc.) of participants across groups, as previously recommended (Baker et al., 2010; Eysenbach et al., 2011). A notable implementation of this recommendation can be seen in Ter Stal et al. (2021). Future studies should not only measure these variables before randomization but also clearly articulate the methods used for generating random allocation sequences, thereby strengthening the research design’s integrity.
- Future research should prioritize the use of reliable measurement methods. Although a significant proportion of the studies we reviewed used reliable methods, some did not report Cronbach’s alpha, a crucial indicator of instrument reliability. This oversight makes it challenging to ascertain whether the instruments accurately measured the intended variables. To ensure methodological rigor, future research must include Cronbach’s alpha to confirm the reliability of their measurement tools, as advised by Kraemer et al. (2002) and Tavakol and Dennick (2011). A good practice is illustrated in a study rated with a moderate methodological quality (Wang et al., 2021), which reports reliability coefficients for each instrument used. Furthermore, our review identified a common issue: the UVE was often measured using single-item indicators, such as “the ECA is attractive,” utilized in a substantial number of studies. This approach may not fully capture the construct’s complexity (Gogol et al., 2014). Also, few studies included a variety of data collection methods. Additionally, only a limited number of studies employed diverse data collection methods. Future investigations should adopt a more comprehensive approach to measuring the UVE, incorporating both subjective and objective measurements, such as physiological or behavioral assessments, to provide a more complete understanding of the UVE.
- To mitigate the risk of exaggerated findings from extreme comparisons, incorporating a neutral condition is advisable whenever possible. For instance, when assessing the impact of the facial emotions of the ECAs on the UVE, it’s beneficial to include scenarios where the ECAs display happiness, sadness, and a neutral face, as presented in Ter Stal et al. (2021). Future studies should clearly state the design, and if it is the case, the number of conditions or the number of measurements across time.
- Future research should meticulously present the procedural details of interventions to enable replication. An exemplary model of this is found in Volante et al. (2016), which thoroughly describes the interaction stages with an ECA used for aviation safety training, covering the introduction, demonstration, practice, and final feedback phases, along with the content conveyed by the ECA in different experimental scenarios. Additionally, our review found a lack of reports on unexpected incidents during interactions between participants and ECAs. Any unexpected event should be reported (Baker et al., 2010).
- Future studies should incorporate a participant flow diagram, a component absent in all reviewed studies. The inclusion of such a diagram is strongly recommended to enhance transparency around data collection methods, offering a clear visual representation of participant progression through the study phases (Rouse et al., 2008).
- When possible, future studies should include qualitative feedback from participants on the advantages and weaknesses of interacting with ECAs. An illustrative case is provided in a study (Volante et al., 2016), where interviews with participants revealed that a significant portion raised concerns about data privacy in their interaction with ECAs. This approach of collecting participant insights is instrumental in uncovering valuable perspectives that could inform enhancements in ECA interaction design.
- Future research should clearly distinguish between pre-specified and exploratory analyses. In the studies reviewed, it was frequently ambiguous whether the analyses had been established before or after data collection. Remarkably, only one study (Volante et al., 2016) had been pre-registered, a practice that clarifies which analyses are confirmatory and which are exploratory, thereby lending greater credibility to the conclusions (Logg and Dorison, 2021). Pre-registration, using platforms like the Open Science Framework, is strongly recommended. Moreover, there’s a pressing need for the use of advanced statistical methods to explore predictors, mediators, and moderators. Delving into these complex analyses can provide crucial insights into customizing interactions to meet individual preferences and needs, thus significantly improving the efficacy and effectiveness of ECAs. There’s a growing need for the application of advanced statistical techniques, especially in the investigation of predictors, mediators, and moderators. Using such analyses could significantly aid in understanding how to design personalized interactions that cater to individual needs, thereby enhancing the overall efficacy and effectiveness of ECAs.

## Conclusion

This systematic review focused on the UVE in user-ECA interactions. However, most studies primarily focused on attractiveness, overlooking the need for a more comprehensive evaluation that includes not only attractiveness but also uncanniness and anthropomorphism. A balanced assessment of all three factors is essential for a deeper understanding of the UVE in ECA design.

Based on the included studies, our findings reveal that among *users*, younger individuals, females, and those with a high openness to new experiences generally perceive ECAs as more attractive. In terms of *ECA features*, customizable agents that are female, and exhibit high levels of extraversion were found to be more attractive. ECAs that exhibit emotional responses congruent with the interaction scenario are viewed more favorably, while a mismatch can lead to perceptions of uncanniness. Moreover, when ECAs are designed to resemble famous figures, precision in meeting user expectations is crucial to prevent uncanniness.

However, it’s noteworthy that the overall methodological quality of the studies examined ranged from weak to moderate according to criteria from the EPHPP instrument. We have proposed methodological strategies for improving user-agent interactions, including the adoption of reliable measurement methods and clear differentiation between pre-defined and exploratory analyses.

To increase the attractiveness of the ECAs, we suggest that ECAs should incorporate features like reflective listening and the capacity to adjust their discourse, facial expressions, and body movements in response to the user’s emotional expressions. Future research is urged to adhere to the recommendations outlined and undertake further investigations to validate and expand upon these initial findings. An exciting avenue for future exploration is the development of ECAs with distinct personalities, expressed through speech, facial expressions, gestures, and eye gaze. Tailoring an ECA’s personality traits to match the user’s personality has the potential to make ECAs more relatable and engaging, thereby reducing uncanniness and increasing user acceptance.

## Data Availability

The original contributions presented in the study are included in the article/Supplementary materials, further inquiries can be directed to the corresponding author.

## Footnotes

^1^ http://www.crd.york.ac.uk/PROSPERO

^2^ https://www.prisma-statement.org/

^3^ https://www.ephpp.ca/quality-assessment-tool-for-quantitative-studies/
