# Supplementary material for: The uncanny valley effect in embodied conversational agents: a critical systematic review of attractiveness, anthropomorphism, and uncanniness
Source: Front Psychol. 2025 Sep 18;16:1625984. doi: 10.3389/fpsyg.2025.1625984 (PMC12493983; doi:10.3389/fpsyg.2025.1625984)
Supplement: Supplementary file 1 [file Supplementary_file_1.docx]

**Appendix 1**

**The list of studies included in the systematic review is arranged alphabetically:**

| **1** | Appel, J., von der Pütten, A., Krämer, N. C., & Gratch, J. (2012). Does userity matter? Analyzing the importance of social cues and perceived agency of a computer system for the emergence of social reactions during user-computer interaction. *Advances in User-Computer Interaction*, 2012, 13-13. |
| --- | --- |
| **2** | Bailey, J. O., & Schloss, J. I. (2024). Knowing versus doing: Children's social conceptions of and behaviors toward virtual reality agents. *International Journal of Child-Computer Interaction*, 40, 100647. |
| **3** | Belda-Medina, J., & Calvo-Ferrer, J. R. (2022). Using Chatbots as AI Conversational Partners in Language Learning. *Applied Sciences*, 12(17), 8427. |
| **4** | Buttussi, F., & Chittaro, L. (2019). Humor and fear appeals in animated pedagogical agents: An evaluation in aviation safety education. *IEEE Transactions on Learning Technologies*, 13(1), 63-76. |
| **5** | Conrad, F. G., Schober, M. F., Jans, M., Orlowski, R. A., Nielsen, D., & Levenstein, R. (2015). Comprehension and engagement in survey interviews with virtual agents. *Frontiers in psychology*, 6, 1578. |
| **6** | Creed, C., & Beale, R. (2012). User interactions with an affective nutritional coach*. Interacting with Computers*, 24(5), 339-350. |
| **7** | Falcone, S., Kolkmeier, J., Bruijnes, M., & Heylen, D. (2022). The multimodal EchoBorg: not as smart as it looks*. Journal on Multimodal User Interfaces*, 16(3), 293-302. |
| **8** | Hale, J., & Hamilton, A. F. D. C. (2016). Testing the relationship between mimicry, trust and rapport in virtual reality conversations. *Scientific reports*, 6(1), 35295. |
| **9** | Ham, J., Li, S., Looi, J., & Eastin, M. S. (2024). Virtual humans as social actors: Investigating user perceptions of virtual humans’ emotional expression on social media. *Computers in Human Behavior*, 155, 108161. |
| **10** | Hao, F., Aman, A. M., & Zhang, C. (2024). What is beautiful is good: attractive avatars for healthier dining and satisfaction. *International Journal of Contemporary Hospitality Management*. |
| **11** | Lahav, O., Talis, V., Cinamon, R. G., & Rizzo, A. (2020). Virtual interactive consulting agent to support freshman students in transition to higher education*. Journal of Computing in Higher Education*, 32, 330-364. |
| **12** | Lisetti, C. L., Brown, S. M., Alvarez, K., & Marpaung, A. H. (2004). A social informatics approach to user-robot interaction with a service social robot. *IEEE Transactions on Systems, Man, and Cybernetics*, Part C (Applications and Reviews), 34(2), 195-209. |
| **13** | Luo, L., Weng, D., Ding, N., Hao, J., & Tu, Z. (2023). The effect of avatar facial expressions on trust building in social virtual reality. *The Visual Computer*, 39(11), 5869-5882. |
| **14** | Min, Q., Sun, H., Wang, X., & Zhang, C. (2024). How do avatar characteristics affect applicants' interactional justice perceptions in artificial intelligence‐based job interviews?. *International Journal of Selection and Assessment*. |
| **15** | Neumann, I., Käthner, I., Gromer, D., & Pauli, P. (2023). Impact of perceived social support on pain perception in virtual reality. *Computers in Human Behavior*, 139, 107490. |
| **16** | Prendinger, H., & Ishizuka, M. (2001). Let's talk! Socially intelligent agents for language conversation training. *IEEE Transactions on Systems, Man, and Cybernetics-Part A: Systems and Users*, 31(5), 465-471. |
| **17** | Prendinger, H., Becker, C., & Ishizuka, M. (2006). A STUDY IN USERS'PHYSIOLOGICAL RESPONSE TO AN EMPATHIC INTERFACE AGENT*. International Journal of Useroid Robotics*, 3(03), 371-391. |
| **18** | Saad, S. B., & Choura, F. (2022). Effectiveness of virtual reality technologies in digital entrepreneurship: a comparative study of two types of virtual agents. *Journal of Research in Marketing and Entrepreneurship*. |
| **19** | Sajjadi, P., Hoffmann, L., Cimiano, P., & Kopp, S. (2019). A personality-based emotional model for embodied conversational agents: Effects on perceived social presence and game experience of users. *Entertainment Computing*, 32, 100313. |
| **20** | Schouten, D. G., Venneker, F., Bosse, T., Neerincx, M. A., & Cremers, A. H. (2017). A digital coach that provides affective and social learning support to low-literate learners. *IEEE Transactions on Learning Technologies*, 11(1), 67-80. |
| **21** | Song, S. W., & Shin, M. (2022). Uncanny Valley Effects on Chatbot Trust, Purchase Intention, and Adoption Intention in the Context of E-Commerce: The Moderating Role of Avatar Familiarity. *International Journal of User–Computer Interaction*, 1-16. |
| **22** | Ter Stal, S., Jongbloed, G., & Tabak, M. (2021). Embodied Conversational Agents in eHealth: How Facial and Textual Expressions of Positive and Neutral Emotions Influence Perceptions of Mutual Understanding. *Interacting with computers*, 33(2), 167-176. |
| **23** | van Pinxteren, M. M., Pluymaekers, M., Lemmink, J., & Krispin, A. (2023). Effects of communication style on relational outcomes in interactions between customers and embodied conversational agents. *Psychology & Marketing*, 40(5), 938-953. |
| **24** | Volante, M., Babu, S. V., Chaturvedi, H., Newsome, N., Ebrahimi, E., Roy, T., ... & Fasolino, T. (2016). Effects of virtual user appearance fidelity on emotion contagion in affective inter-personal simulations*. IEEE transactions on visualization and computer graphics*, 22(4), 1326-1335. |
| **25** | Wang, H., Gaddy, V., Beveridge, J. R., & Ortega, F. R. (2021). Building an emotionally responsive avatar with dynamic facial expressions in user—computer interactions. *Multimodal Technologies and Interaction*, 5(3), |
| **26** | Yin, J., Wang, S., Guo, W., & Shao, M. (2021). More than appearance: the uncanny valley effect changes with a robot’s mental capacity. *Current Psychology*, 1-12. |
| **27** | Zhang, J., Chen, Q., Lu, J., Wang, X., Liu, L., & Feng, Y. (2024). Emotional expression by artificial intelligence chatbots to improve customer satisfaction: Underlying mechanism and boundary conditions. *Tourism Management*, 100, 104835. |
| **28** | Zheleva, A., Hardeman, J., Durnez, W., Vanroelen, C., De Bruyne, J., Tutu, D. O., ... & Bombeke, K. (2023). The impact of eye gaze on social interactions of females in virtual reality: The mediating role of the uncanniness of avatars and the moderating role of task type. *Heliyon*, 9(10). |
| **29** | Zibrek, K., Kokkinara, E., & McDonnell, R. (2018). The effect of realistic appearance of virtual characters in immersive environments-does the character's personality play a role?. *IEEE transactions on visualization and computer graphics*, 24(4), 1681-1690. |
